# NAIL-MS reveals the repair of 2-methylthiocytidine by AlkB in *E. coli*

**DOI:** 10.1038/s41467-019-13565-9

**Published:** 2019-12-06

**Authors:** Valentin F. Reichle, Dimitar P. Petrov, Verena Weber, Kirsten Jung, Stefanie Kellner

**Affiliations:** 1 Department of Chemistry, Ludwig-Maximilians-University Munich, Butenandtstr. 5-13, 81377 Munich, Germany; 2 Department of Biology, Ludwig-Maximilians-University Munich, Grosshaderner Str. 2-4, 82152 Martinsried, Germany

**Keywords:** Chemical modification, RNA, RNA modification

## Abstract

RNAs contain post-transcriptional modifications, which fulfill a variety of functions in translation, secondary structure stabilization and cellular stress survival. Here, 2-methylthiocytidine (ms^2^C) is identified in tRNA of *E. coli* and *P. aeruginosa* using NAIL-MS (nucleic acid isotope labeling coupled mass spectrometry) in combination with genetic screening experiments. ms^2^C is only found in 2-thiocytidine (s^2^C) containing tRNAs, namely tRNA^Arg^_CCG_, tRNA^Arg^_ICG_, tRNA^Arg^_UCU_ and tRNA^Ser^_GCU_ at low abundances. ms^2^C is not formed by commonly known tRNA methyltransferases. Instead, we observe its formation in vitro and in vivo during exposure to methylating agents. More than half of the s^2^C containing tRNA can be methylated to carry ms^2^C. With a pulse-chase NAIL-MS experiment, the repair mechanism by AlkB dependent sulfur demethylation is demonstrated in vivo. Overall, we describe ms^2^C as a bacterial tRNA modification and damage product. Its repair by AlkB and other pathways is demonstrated in vivo by our powerful NAIL-MS approach.

## Abstract (web summary)

Bacterial tRNA is modified by thiolation of nucleosides. Here the authors identify 2-methylthiocytidine in bacterial tRNA using nucleic acid isotope labeling coupled mass spectrometry. Exposure to methylating agents converts 2-thiocytidine to 2-methylthiocytidine, which is repaired by demethylase AlkB in vivo.

## Introduction

Each Nucleic acid is composed of four canonical nucleobases, which are connected through ribose (RNA) or 2′-O-deoxyribose (DNA) and phosphate. In addition, both DNA and RNA are target of modifying enzymes which introduce methylations or other functionalities. To this day, over 160 modified nucleosides have been identified in RNA from organisms in all domains of life^1^.

Due to the nucleophilic character of the canonical nucleobases, both RNA and DNA are prone to direct methylation. Inside cells these electrophiles comprise molecules such as S-adenosylmethionine (SAM), which is the common methyl-donor for enzymatic methylation reactions but it is also known to directly alkylate nucleic acids^2^. In addition, bacteria use various electrophiles as chemical warheads (e.g., streptozotocin, azaserine or methylenchloride) to harm other bacteria by methylation and alkylation of their macromolecules^3–6^. Thus, bacteria are constantly forced to overcome these alkylating reagents and various adaptive mechanisms can be found. To study the impact of direct methylation, electrophiles such as methyl-methanesulfonate (MMS) or dimethyl sulfate (DMS) are used. Methylation damage of both RNA and DNA is repaired by direct demethylation through the alpha-ketoglutarate dependent dioxygenase AlkB in bacteria^7,8^. So far the methylated aromatic nitrogens of 3-methylcytidine (m^3^C), 1-methyladenosine (m^1^A) and 1-methylguanosine (m^1^G) are reported substrates of AlkB. In addition, the exocyclic amines of N2,N2-dimethylguanosine (m^2,2^G) can be demethylated by AlkB in vitro, which finds application in the RNA modification sequencing technique ARM-Seq^9^. So far, no non-nitrogen methylations have been described as substrates of AlkB in RNA.

In addition to the nucleobase methylations introduced by direct alkylation, cells in all domains of life use SAM for the enzymatic methylation of RNA. The occurrence of these natural methylated nucleosides makes the detection of damage-derived methylated nucleosides challenging. With the application of CD_3_-labeled methionine in a nucleic acid isotope labeling coupled mass spectrometry (NAIL-MS) assay, this discrimination can be achieved and, e.g., enzymatic 7-methylguanosine (m^7^G) and MMS-damage derived m^7^G can be quantified independently^10^. With NAIL-MS, the analysis of alkylation damage of canonical nucleosides was possible in the presence of natural methylated nucleosides. A key finding in this study was that m^7^G damage is as common as m^1^A, which was believed to be the only dominant RNA damage product upon direct methylation in vivo^11^. In addition, NAIL-MS was used in a pulse-chase setup to follow the in vivo demethylation kinetics of m^1^A and m^3^C during MMS recovery^10^. Especially m^3^C is quickly and efficiently (~80%) repaired within nine hours of recovery whereas only 20% of m^1^A damage sites are repaired within the same timeframe^12^. For m^3^C, the demethylation by AlkB is reported in vitro, but so far not in vivo^13^.

While methylation is a major modification of RNA, especially tRNA contains additional and more complex modifications. One group of unique tRNA modifications is the enzymatic thiolation. For example, bacteria thiolate cytidine at position 32 of the tRNA to form 2-thiocytidine (s^2^C). The thiolated nucleosides at position 32 and 37 of tRNA flank the anticodon (position 34–36) allowing for a more flexible anticodon loop and thus improve the binding of the codon during translation. s^2^C is incorporated into bacterial tRNA^Arg^_CCG_, tRNA^Arg^_ICG_, tRNA^Arg^_UCU_ and tRNA^Ser^_GCU_ by the thiotransferase TtcA at position 32. Its effects on tRNA structure and bacterial translation has been addressed in several studies^14,15^.

Here, through the use of genetic and analytical tools, we identify and confirm the structure of 2-methylthiocytidine as an endogenous modification in bacterial tRNA. In accordance with the nomenclature provided by the RNA community^1^ it is abbreviated as ms^2^C. The new modification is present in the same tRNA isoacceptors as its precursor modification s^2^C, but only 1% of all s^2^C modified tRNAs are methylated to ms^2^C. Due to the low abundance of ms^2^C in vivo and its formation by SAM in vitro, ms^2^C might present an endogenous tRNA lesion. During exposure of bacteria to the natural antibiotic streptozotocin and the alkylating reagent MMS, up to 50 % of the s^2^C modified tRNAs become methylated and ms^2^C is formed. Thus, ms^2^C is an additional damage product of bacterial RNA and of comparable abundance to the main methylation products m^1^A and m^7^G in tRNA^10,11^. With our unique NAIL-MS approach, we observe a fast and efficient repair of ms^2^C and regeneration of s^2^C in the damaged tRNAs. With NAIL-MS, we identify high demethylation activity of AlkB towards ms^2^C and m^3^C, and only slow demethylation of the described substrate m^1^A. In addition, we observe s^2^C from rethiolated cytidine after dethiomethylation by an AlkB independent mechanism. Overall, NAIL-MS is a tool which is useful for the identification of nucleoside structures and allows deeper insights into RNA demethylation mechanisms in vivo.

## Results

### 2-methylthiocytidine is a bacterial tRNA modification

**Fig. 1:**
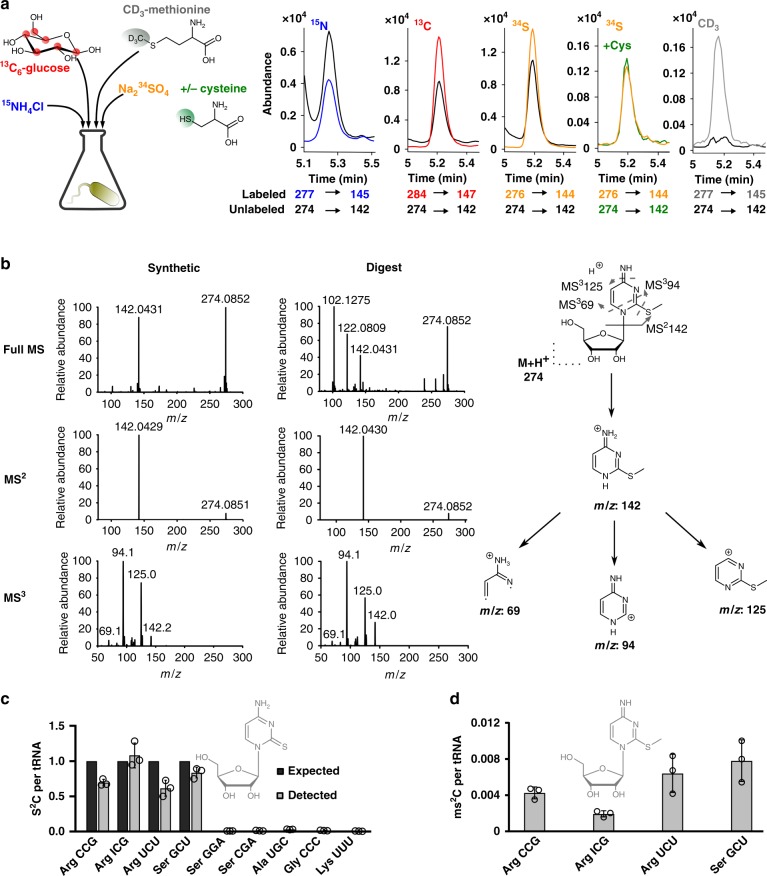
Verification of 2-methylthiocytidine (ms^2^C) structure and distribution in bacterial tRNA isoacceptors. **a** Different isotope labeling in bacteria. Co-elution of the synthesized ms^2^C nucleoside (black) and ms^2^C from [^15^N], [^13^C] or [^34^S] labeled bacteria and products of metabolic labeling with [^32^S]-cysteine in [^34^S] medium and CD_3_-methionine labeling. The respective isotope is indicated above each chromatogram. **b** High resolution mass spectrometry and fragmentation of ms^2^C by direct injection of the synthetic standard or the natural bacterial digest and collision induced dissociation. **c** Expected (black)^1^ and experimentally detected (gray) abundance of s^2^C per respective tRNA isoacceptor. **d** Experimentally detected abundance of ms^2^C per respective s^2^C containing tRNA isoacceptor. All experiments are from *n* = 3 biol. replicates, error bars reflect standard deviation. Source data of (**c**) and (**d**) are provided as a Source Data file.

With the goal of discovering novel modified nucleosides in bacterial tRNA, *Escherichia coli* and *Pseudomonas aeruginosa* were grown in differentially stable isotope labeled growth media as recently reported^16,17^. The total tRNA were purified and analyzed by mass spectrometry. After data evaluation we found a promising candidate eluting at 5.2 min with an *m/z* of 274 from unlabeled tRNA, an *m/z* of 284 from [^13^C]-labeled tRNA, an *m/z* of 277 from [^15^N]-labeled tRNA and an *m/z* of 276 from [^34^S]-labeled tRNA (Fig. 1a, Supplementary Table 1). The sum formula of the nucleobase is C_5_H_*x*_N_3_O_*y*_S_1_ (Supplementary Fig. 1) and the nucleoside is thus C_10_H_*X*_N_3_O_*X*_S_1_. Through the addition of the heavy labeled amino acid CD_3_-methionine to the growth medium, we predicted the presence of a single methyl-group in the nucleoside candidate. Aiming at clarification of the analyte’s structure, we screened *E. coli* knockouts for its absence. TtcA is responsible for 2-thiolation of cytidine and in the Δ*ttcA* strain, the *m/z* 274 signal is lost alongside 2-thiocytidine (s^2^C) (Supplementary Fig. 2a, Supplementary Tables 2 and 3)^18^. The mass difference of the novel nucleoside (*m/z* 274) and s^2^C (*m/z* 260) is 14 Dalton, which is a strong indicator of methylation of s^2^C.

Due to the high nucleophilicity of thiols, we predicted that both the chemical synthesis and biosynthesis of 2-methylthiocytidine (ms^2^C) is feasible, and thus the methylation reaction at position 2 of s^2^C was performed. Interestingly, the synthetic route was already described in 2010, when the Suzuki lab^19^ used 2-methylthiocytidine as an intermediate of agmatidine synthesis. We followed their synthetic route under milder conditions and received pure ms^2^C in 28% yield (Supplementary Figs. 2b and 3). We mixed the synthesized ms^2^C with the heavy isotope labeled tRNA digests and observed perfect co-elution for the synthesized compound and its native isotopologues (Fig. 1a). Additional high resolution MS, MS² and MS³ spectra of the synthesized product and of native tRNA confirmed the structure of the novel nucleoside to be ms^2^C in *E. coli* (Fig. 1b) and *P. aeruginosa* (Supplementary Fig. 4).

With the synthetic standard of ms^2^C and an internal standard produced by metabolic isotope labeling^20^, we could now quantify the absolute abundance of ms^2^C in specific tRNAs from the unstressed *E. coli* BW25113 wild-type strain (WT). The precursor of ms^2^C, s^2^C is reported in three of the five tRNA^Arg^ isoacceptors and one of the five tRNA^Ser^ isoacceptors^1,14,21^. Therefore, we designed DNA probes (Supplementary Table 4) against tRNAs with a reported s^2^C, namely tRNA^Arg^_CCG_, tRNA^Arg^_ICG_, tRNA^Arg^_UCU_ and tRNA^Ser^_GCU_, and purified these isoacceptor tRNAs from total tRNA^22^. As a control we decided to isolate tRNAs with C32 (tRNA^Ser^_GGA_, tRNA ^Lys^_UUU_), a tRNA with 2’-O-methylcytidine at position 32 (Cm32, tRNA^Ser^_UGA_) and tRNAs without a C32, tRNA^Gly^_CCC_ and tRNA^Ala^_UGC_. As expected, we found s^2^C in tRNA^Arg^_CCG_, tRNA^Arg^_ICG_, tRNA^Arg^_UCU_ and tRNA^Ser^_GCU_ (Fig. 1c). The complete modification profile of these tRNAs is shown in Supplementary Table 5 and Supplementary Fig. 5. ms^2^C was also found in these s^2^C-containing tRNA isoacceptors, however according to our data only 0.2–1.0% of actually s^2^C-modified tRNAs contain ms^2^C.

For exploration of the biosynthetic pathway, we quantified the abundance of ms^2^C in total tRNA from the *E. coli* WT and various isogenic knockout strains (Supplementary Table 2) in glucose-containing M9 minimal medium (Supplementary Fig. 6) and LB medium (Supplementary Fig. 7). In total tRNA from WT *E. coli* grown in M9 we found 0.08 s^2^C per average tRNA, and only ~0.00032 ms^2^C. In the Δ*ttcA* mutant no s^2^C and ms^2^C were detectable (corresponding to less than 0.00005 modifications per tRNA). We observed decreased ms^2^C formation in various knockout strains grown in M9 medium but not in LB medium. To exclude an involvement of these enzymes in ms^2^C formation, we analyzed *E. coli* strains that overexpress tRNA modifying enzymes (Supplementary Fig. 7 and Supplementary Table 6). This confirmed that none of the investigated enzymes is involved in ms^2^C biosynthesis. The overall low abundance and high fluctuation of ms^2^C in unstressed bacteria hints towards its nature as an endogenous tRNA lesion. Indeed, in vitro experiments showed the formation of ms^2^C from s^2^C in the presence of SAM and MMS (Supplementary Fig. 8).

### 2-thiocytidine is a main substrate of MMS alkylation in vivo

**Fig. 2:**
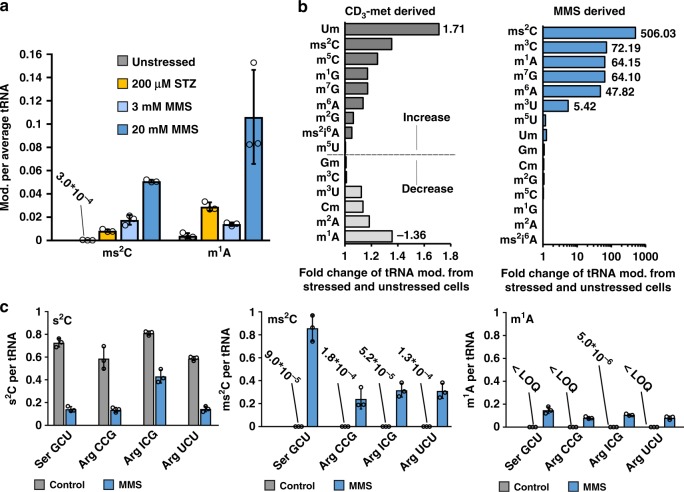
Absolute abundance of modified nucleosides in total tRNA and tRNA isoacceptors after methylation stress. **a** Abundance of mod. (ms^2^C and m^1^A) per average tRNA after 0 mM (unstressed, gray), 200 µM Streptozotocin (STZ, natural methylating agent, 50% lethality, orange), 3 mM MMS (no lethality, light blue) and 20 mM MMS (50% lethality, dark blue). **b** Impact of MMS exposure (unstressed vs. 20 mM stressed) on *E. coli* total tRNA modifications (mod.). The origin of the methylated tRNA modifications is displayed in separate plots. Left: impact of MMS exposure on CD_3_-met (met = methionine) derived methylation displayed as the fold change ratio of stressed-to-unstressed. Right: impact of MMS exposure on directly methylated nucleosides (MMS derived methylation) displayed as the fold change of stressed-to-unstressed. **c** Absolute abundance of s^2^C (left), ms^2^C (middle) and m^1^A (right) in various tRNA isoacceptors without (gray) and with (blue) 20 mM MMS exposure. In the negative control (without MMS) *E. coli* were grown in ^13^C medium (gray bars, control) and the MMS exposed bacteria in non-labeled (blue bars, MMS) media. Co-purification of tRNA isoacceptors was done in a comparative NAIL-MS experiment as detailed in the text. All experiments are from *n* = 3 biol. replicates and error bars reflect standard deviation. Source data are provided as a Source Data file.

Intrigued by the possibility that s^2^C is methylated in vivo by naturally occurring methyl donors like SAM, we were wondering about its methylation during alkylation stress. We exposed WT *E. coli* to the LD_50_ dose of MMS (20 mM), non-lethal MMS doses (3 mM and 0.5 mM) and the LD_50_ dose of the natural antibiotic streptozotocin (STZ, 200 µM) in M9 medium (Supplementary Fig. 9). The total tRNA was isolated for absolute quantification by LC-MS/MS (Supplementary Table 7). The results for the main RNA damage product m^1^A and ms^2^C are shown in Fig. 2a. We observed ~0.1 m^1^A per tRNA and ~0.05 ms^2^C per tRNA in the 20 mM MMS exposed bacteria. With the non-lethal dose of 3 mM MMS, the formation of damage is reduced to 0.014 m^1^A and 0.018 ms^2^C per tRNA. With low doses of alkylating agent, the s^2^C damage was more prominent in total tRNA than the adenosine damage. The absolute abundance of other modified nucleosides is shown in Supplementary Figs. 10 and 11. STZ methylated both adenosine (0.03 m^1^A/tRNA) and s^2^C (0.008 ms^2^C/tRNA). Overall, ms^2^C formed readily during alkylation stress and in comparable extent to m^1^A.

While m^1^A is known to be a direct methylation product, the increase in ms^2^C might be caused by either direct methylation or SAM-derived enzymatic methylation. To elucidate the origin of the methylation in ms^2^C under MMS stress, we used nucleic acid isotope labeling coupled mass spectrometry (NAIL-MS) to discriminate direct and SAM-derived methylation during MMS stress in vivo. Adapted from our recently established assay^10^, we cultured *E. coli* WT bacteria in the presence of CD_3_-methionine to allow complete CD_3_-labeling of all SAM dependent methylated nucleosides. One aliquot of these bacteria was exposed to the LD_50_ dose of MMS, while the other remained unstressed. After 1 h of MMS exposure the tRNA was purified and analyzed by LC-MS/MS. Enzymatically methylated nucleosides carry a + 3 label whereas the directly MMS methylated nucleosides are unlabeled and thus the two species are distinguishable by mass spectrometry. Absolute quantification of the enzymatically and directly methylated nucleosides in the unstressed and stressed cells revealed the abundance of methylated nucleosides in the samples. The fold changes between the stressed and unstressed samples were calculated and plotted in Fig. 2b. We observed only little adaptation of enzymatically introduced tRNA modifications under these growth conditions. The strongest increase was 1.7 fold, observed for Um (2′-O-methyluridine) from total tRNA. In both the stressed and unstressed samples, ms^2^C was again found to be labeled through CD_3_-methionine dependent pathways, with a slightly higher abundance in the stressed samples. This small increase in CD_3_-methylated ms^2^C does not reflect its substantial abundance in Fig. 2a. In contrast to the SAM dependent methylations, we found a high increase for the known products of direct methylation, namely m^3^U (5 fold), m^6^A (47 fold), m^7^G (64 fold) m^1^A (64 fold) and m^3^C (74 fold)^10^. However, the highest increase was for the product of direct s^2^C methylation, ms^2^C, which was over 500 fold increased in the stressed bacteria compared to the unstressed (absolute quantities in Supplementary Fig. 12). Thus, we prove that the ms^2^C observed in Fig. 2a originated from direct s^2^C methylation by MMS.

The previous experiments were done with total tRNA, but only four tRNA isoacceptors carry s^2^C. To study the impact of direct methylation on these s^2^C-containing tRNAs, we performed a comparative NAIL-MS experiment. For this purpose, the unstressed *E. coli* are grown in ^13^C_6_-glucose labeled medium and the MMS exposed bacteria (LD_50_) in unlabeled medium. After cell harvesting, the unstressed and stressed bacteria were mixed to avoid purification biases, as recently suggested^12^. From the resulting co-purified total tRNA, the tRNA isoacceptors Ser^GCU^, Arg^CCG^, Arg^ICG^ and Arg^UCU^ were isolated, digested and subjected to LC-MS/MS analysis. The mass spectrometer was set to distinguish the ^13^C-labeled nucleosides (unstressed bacteria) from the unlabeled nucleosides (stressed bacteria) and thus the abundance of each nucleoside can be determined (Supplementary Figs. 13 and 14, Supplementary Tables 8 and 9). On average, we found 0.8 s^2^C per respective unstressed tRNA, but after MMS exposure, the abundance of s^2^C drops substantially (Fig. 2c, left). For tRNA Ser^GCU^, Arg^CCG^ and Arg^UCU^ we found a 4-fold decrease to <0.2 s^2^C per tRNA. tRNA^Arg^_ICG_ was the only exception with a ~2-fold decrease to around 0.4 s^2^C per tRNA. As expected, the abundance of ms^2^C was increased in the stressed samples, which indicated that the lost s^2^C was directly methylated to ms^2^C in these tRNAs (Fig. 2c, middle). In all purified tRNAs, the ms^2^C abundance was higher than the m^1^A abundance (Fig. 2c, right).

This data demonstrates that s^2^C is a better substrate of the methylation agent MMS than adenosine.

### 2-methylthiocytidine may influence translation

**Fig. 3:**
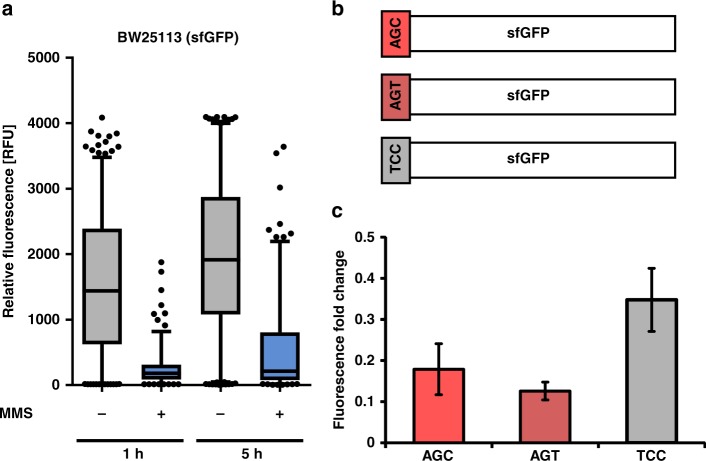
Impact of s^2^C methylation on translation. **a** Long-term effects of 20 mM MMS stress on sfGFP synthesis in *E. coli* WT cells. Single cell fluorescence was determined for a minimum of 300 *E. coli* cells producing sfGFP under control of the L-arabinose inducible promoter (pBAD24). Fluorescence was measured 1 and 5 h after addition of arabinose to unstressed cells or cells pre-exposed to 20 mM MMS stress for 1 h. (box plots represent the 5-95% percentile) **b** Illustration of the used sfGFP constructs. The serine on amino acid position 2 of the used sfGFP variant was encoded by different codons: AGC, AGT and TCC. **c** Influence of codons on sfGFP synthesis after exposure of cells to MMS stress. Fluorescence was measured after 1 h of induction with arabinose. The fold change was determined by dividing the mean fluorescence of stressed cells (20 mM MMS) by the mean fluorescence of unstressed cells. All experiments are from *n* = 3 biol. replicates and error bars reflect standard deviation. Source data are provided as a Source Data file.

The s^2^C containing tRNAs are major targets of direct methylation by MMS. s^2^C is located at position 32 where it fulfils important roles in translation of the serine codons AGU and AGC by tRNA^Ser^_GCU_ and the arginine codons CGG (tRNA^Arg^_CCG_), AGA (tRNA^Arg^_UCU_) and CGC, CGU and CGA (tRNA^Arg^_ICG_)^14^. The methylation of s^2^C and formation of ms^2^C might result in electronic and electrostatic shifts of the nucleobase, which potentially changes the properties of the whole anticodon loop of the affected tRNAs. Thus translation of the respective serine and arginine codons might be influenced by the methylation of s^2^C. To address this question, the effect of MMS on the synthesis of superfolder green fluorescent protein (sfGFP) in *E. coli* WT was determined. We decided to study sfGFP (for sequence and used primers see Supplementary Table 10), which contains one AGC (at position 2) and one AGT codon for serine, five GCU, two CGC and one CGG codon for arginine. 10 codons are translated by s^2^C modified tRNAs, which will be damaged to carry ms^2^C under MMS stress. Thus the translation of sfGFP could be effected by MMS due to the s^2^C methylation. Expression of *sfgfp* was under control of the P_BAD_ promoter, and unstressed or stressed (exposures to 20 mM MMS for 1 h) cells of the mid-log growth phase were induced by arabinose. Fluorescence intensity of single *E. coli* cells of strain BW25113 was determined after 1 and 5 h (Supplementary Fig. 15). In the presence of 3 mM MMS, fluorescence was significantly lower under acute stress (1 h), but cells recovered after 5 h indicated by the increase in fluorescence. Similar results are seen for cells after exposure to 20 mM MMS (Fig. 3a). We mutated a single codon in the *sfgfp* gene to replace the AGC codon for serine with AGT and TCC (Fig. 3b). While AGC and AGT are read by the same s^2^C modified tRNA^Ser^_GCU_, the TCC codon is read by tRNA^Ser^_GGA_, which is not s^2^C modified and thus the anticodon is not affected by the MMS stress (see Fig. 1c). We tested the influence of these codons (AGC, AGT, TTC) at position 2 in sfGFP on the fluorescence in the WT strain. Fluorescence of stressed cells producing the AGT- and AGC-variants of sfGFP is more affected in comparison to the TCC construct (Fig. 3c). At the used MMS doses, we previously observed about 80% ms^2^C modified tRNA Ser^GCU^ while only 20% were s^2^C modified (Fig. 2c). Thus we see a direct correlation of ms^2^C abundance and decreased translational efficiency for the respective codons. Moreover, translation of the AGT-containing sfGFP was slightly more affected by the methylating agent than the AGC-containing construct. These results indicate a negative effect of ms^2^C on translation.

### 2-methylthiocytidine is efficiently repaired by AlkB

**Fig. 4:**
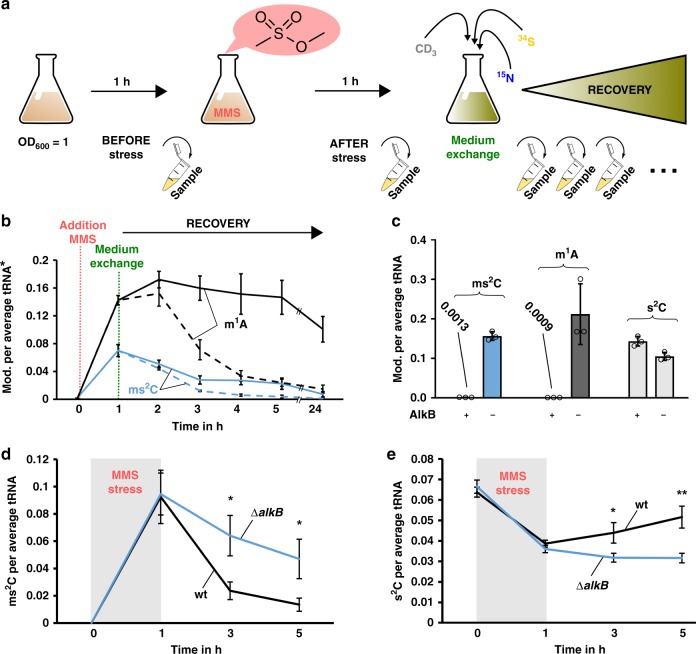
Principle and results of pulse-chase NAIL-MS experiments to determine the repair of ms^2^C in vivo. **a** Principle of a pulse-chase NAIL-MS experiment. The bacteria are grown in unlabeled (n.l.) media before and after exposure to MMS (structure shown). After 1 h MMS exposure, the media is removed and fresh, [^15^N], [^34^S] and [CD_3_]-methionine containing media is added. Samples are drawn during the recovery time for tRNA isolation. **b** Formation of the nucleoside damage product ms^2^C (blue) and m^1^A (black) per average tRNA after 20 mM MMS exposure. *Dashed lines: abundance of mod. (ms^2^C and m^1^A) per all tRNAs (sum of original, unlabeled and new, [^15^N]-labeled transcripts). Solid lines: abundance of mod. (ms^2^C and m^1^A) per original tRNA. **c** Abundance of mod. (ms^2^C in blue, m^1^A in dark gray and s^2^C in light gray) per tRNA incubated with purified AlkB in vitro. The substrate tRNA is isolated from *E. coli* bacteria exposed to 20 mM MMS. **d** ms^2^C abundance of *E. coli* WT (black) in comparison to *alkB* deficient *E. coli* (Δ*alkB*, blue) after MMS stress (20 mM, 1 h) and during recovery in a pulse-chase NAIL-MS experiment, as described in a) and b). **e** s^2^C abundance of *E. coli* WT (black) in comparison to *alkB* deficient *E. coli* (Δ*alkB*, blue) after MMS stress (20 mM, 1 h) and during recovery. All experiments are from n = 3 biol. replicates and error bars reflect standard deviation. *p*-values from student t-test (equal distribution, two-sided): *p < 0.05 and **p < 0.01. Source data are provided as a Source Data file.

The ms^2^C damage has a potential effect on the translation of *E. coli*. It is highly probable that the methylation damage is recognized and repaired by the bacteria to adapt to the stress. This repair is either possible by direct demethylation by AlkB as shown for m^1^A^23^ or by tRNA degradation. To address the question whether ms^2^C is repaired and which mechanism dominates, we designed a pulse-chase assay involving stable isotope labeling. Figure 4a shows the principle of the assay. The goal of this assay is to discriminate the damaged tRNAs from the tRNAs transcribed during the recovery. Thus, we can follow the fate of the damaged tRNAs and their nucleosides independently from dilution by transcription. For this purpose, cells were grown in media containing only [^14^N]- and [^32^S]-nutrients. Consequently, the RNA was completely labeled with [^14^N] and all s^2^C have a [^32^S] label (original s^2^C), *e.g. m/z* (s^2^C) 260. All methylated nucleosides had a CH_3_ mark (e.g., original m^1^A *m/z* 282). In this medium, the cells were exposed to MMS (LD_50_) and s^2^C was converted to ms^2^C and, e.g., A to m^1^A. After exposure, MMS was removed by exchanging the media with heavy-isotope media. During the following recovery period, newly transcribed RNA were [^15^N] labeled, newly methylated nucleosides were [CD]_3_ labeled and new s^2^C had a [^34^S] label (new s^2^C, *m/z* 265 and new m^1^A, *m/z* 290).

The dashed line in Fig. 4b, represents the decrease of m^1^A if referenced to the sum of all tRNAs (original + new transcripts). The fast decrease is caused by dilution due to ongoing tRNA transcription. In the past, this decrease could be misinterpreted as active tRNA demethylation, but with NAIL-MS we can follow the fate of modified nucleosides inside living cells by excluding transcription processes. By referencing m^1^A abundance to the original tRNAs, we saw a slow but steady decrease of m^1^A in the damaged tRNAs over time which reflects true demethylation (Fig. 4b, solid line). For ms^2^C, we saw a fast and nearly complete repair over the same observation period (Fig. 4b). This decrease was not caused by increased tRNA degradation as MMS exposed bacteria showed the same original-tRNA dilution pattern as unstressed bacteria (Supplementary Fig. 16). We isolated tRNAs Ser^GCU^ and Arg^ICG^ from the NAIL-MS pulse-chase experiments, and for these damaged tRNAs we saw no increased degradation either (Supplementary Fig. 17). Thus we conclude that the removal of ms^2^C from damaged tRNA is not caused by tRNA degradation.

m^1^A is known to be demethylated by AlkB^13,23^. To test whether AlkB also demethylates ms^2^C to s^2^C, we isolated total tRNA from MMS exposed bacteria and tested the AlkB activity in vitro. After addition of AlkB to the damaged tRNA, we could no longer detect ms^2^C and m^1^A in the tRNA (Fig. 4c). Instead, we could observe an increase of s^2^C, which indicates that AlkB demethylates ms^2^C to s^2^C. This finding is rather surprising as, until now, only nitrogen demethylation was observed for AlkB but never sulfur demethylation^11,23^.

To verify that AlkB demethylates ms^2^C containing tRNAs in vivo, we decided to repeat the NAIL-MS pulse-chase assay in an *alkB* deficient *E. coli* strain. After exposure to 20 mM MMS, we observed a fast decrease of ms^2^C in the WT strain and a slower decrease in the isogenic Δ*alkB* mutant (Fig. 4d). Interestingly, we observed an increase of s^2^C in the WT strain but not in the Δ*alkB* strain (Fig. 4e). Our data indicates that a portion of ms^2^C is demethylated by AlkB under recovery of the original [^32^S]-s^2^C. The other portion of ms^2^C seems to be lost by an AlkB-independent mechanism. For m^3^C we also observed an AlkB dependent demethylation, but surprisingly not for m^1^A (Supplementary Fig. 18). We repeated the experiment in the presence of 3 mM MMS and 0.5 mM MMS to ensure the viability of the cells. 3 mM MMS corresponds to 20% lethality in the Δ*alkB* strain (Supplementary Fig. 19) and no lethality in the WT strain. Directly after 3 mM MMS exposure, the abundance of ms^2^C, m^1^A and m^3^C per original tRNA was comparable in the WT and Δ*alkB* strain (Supplementary Fig. 20). After 2 h of recovery, the abundance of ms^2^C, m^1^A and m^3^C was more reduced in the WT strain compared to the Δ*alkB* mutant. This indicates repair of ms^2^C by AlkB in vivo as observed in Fig. 4d. For 0.5 mM MMS, we saw an AlkB independent reduction of m^1^A, m^3^C, and ms^2^C of comparable extent. On tRNAs with a damage abundance below a threshold level, AlkB appeared to be uninvolved in repair.

In summary, we demonstrate that ms^2^C damaged tRNAs are yet undescribed substrates of AlkB, both in vitro and in vivo.

### Dethiomethylation as an alternative repair pathway

The in vitro as well as the in vivo analyses of AlkB convinced us of the role of AlkB as key player in ms^2^C demethylation. Based on our data shown in Fig. 4, we assume direct demethylation by AlkB, which results in the recovery of [^32^S]-s^2^C.

**Fig. 5:**
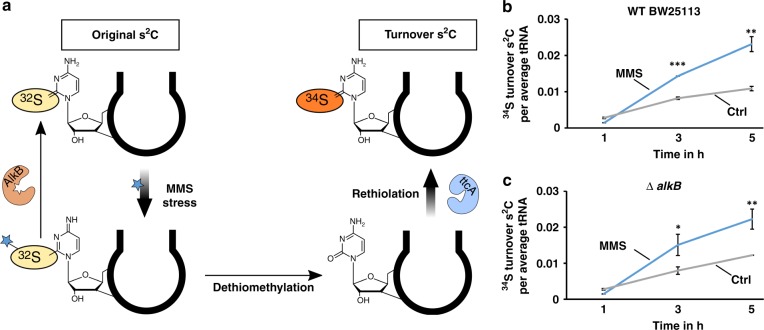
Pulse-chase NAIL-MS analysis of turnover s^2^C after 20 mM MMS exposure in WT BW25113 and *E. coli* Δ*alkB* knockout strain. **a** Concept of turnover s^2^C generation and its detection by NAIL-MS. **b** Abundance of [^34^S] turnover s^2^C in WT BW25113 during recovery after MMS stress (20 mM) and unstressed control. **c** Abundance of [^34^S] turnover s^2^C in the Δ*alkB* strain during recovery after MMS stress (20 mM) and unstressed control. All experiments are from *n* = 3 biol. replicates and error bars reflect standard deviation. *p*-values from student t-test (equal distribution, two-sided): *p < 0.05, **p < 0.01 and ***p < 0.001. Source data are provided as a Source Data file.

Chemically, the thiomethyl group is an acceptable leaving group in the presence of nucleophiles such as water. Dethiomethylation might occur inside the cell and would result in the formation of canonical cytidine. In theory, this cytidine should then be a substrate of TtcA and a rethiolation to s^2^C would result (Fig. 5a). Such an s^2^C turnover event is observable in vivo with our pulse-chase NAIL-MS experiment. During the design of the experiment, we chose two media with different sulfur isotopes. The stress medium contains the sulfur-32 isotope and thus all s^2^C of original tRNAs is [^32^S]-s^2^C with an *m/z* of 260. The recovery medium contains no sulfur-32, but sulfur-34 instead. Thus, s^2^C in new transcripts is not only three units heavier (nitrogen-15) but five units ([^15^N_3_/^34^S]-s^2^C, *m/z* 265). In case of the imagined scenario of spontaneous dethiomethylation of ms^2^C into cytidine followed by rethiolation into s^2^C, only sulfur-34 is available and thus the original [^32^S]-s^2^C would turn into [^34^S]-s^2^C with an *m/z* of 262. The original [^32^S]-s^2^C produced by AlkB demethylation and the hypothetical turnover [^34^S]-s^2^C formed after dethiomethylation/rethiolation can be distinguished by mass spectrometry (Supplementary Table 11).

The result for the unstressed and 20 mM MMS treated WT strain is shown in Fig. 5b. Immediately after the stress, the number of turnover [^34^S]-s^2^C was comparable and rose over time. The increase of [^34^S]-s^2^C in unstressed bacteria was most likely caused by regular s^2^C biosynthesis into original tRNAs, which were not fully mature at the time of medium exchange. The presence of only [^34^S] at these later time points led to the formation of [^34^S]-s^2^C in the control bacteria. In contrast, MMS exposure led to more [^34^S]-s^2^C. To test involvement of AlkB in the formation of turnover [^34^S]-s^2^C, we plotted the [^34^S]-s^2^C abundance observed in the Δ*alkB* strain (Fig. 5c). Here, we saw an increase in turnover s^2^C, which was comparable to the turnover in the WT strain. We thus conclude that the turnover is not a consequence of the AlkB repair process.

The increase in turnover s^2^C can be explained by dethiomethylation of ms^2^C followed by rethiolation of the formed cytidine. Currently it is unclear, whether the dethiomethylation occurs spontaneously after a nucleophilic attack of water or by an enzymatic pathway. From a chemical perspective, we believe that spontaneous dethiomethylation is the most likely.

## Discussion

In this study, we identified s^2^C as a substrate of direct methylation in bacterial tRNAs, and the mechanisms of the subsequent repair by AlkB. We initially identified the new structure of the methylated nucleoside in total tRNA of *E. coli* and *P. aeruginosa*. The structure of the ms^2^C (2-methylthiocytidine) nucleoside was confirmed after synthesis by various analytical tools including fragmentation patterns in high resolution mass spectrometry. Methylated derivatives of other thiolated nucleosides like s^4^U or mnm^5^s^2^U might also form endogenously, but were not detected in our nucleoside discovery experiments.

ms^2^C was found at position 32 of tRNA^Arg^_ICG_, tRNA^Arg^_CCG_, tRNA^Arg^_UCU_ and tRNA^Ser^_GCU_. Only 1% of the s^2^C modified tRNA isoacceptors carry ms^2^C under unstressed growth conditions. The endogenous formation of ms^2^C was not found to be connected to common tRNA modifying enzymes. At this stage we cannot determine whether the endogenous, but low abundant, ms^2^C modification of bacterial tRNA is introduced enzymatically or by direct methylation through, e.g., SAM. For low abundant nucleoside modifications it is often an open question whether they are lesions or functional entities^24^.

Intrigued by the observation that ms^2^C forms naturally in the presence of the methyl donor SAM, we were wondering whether ms^2^C is formed by direct methylation during alkylation stress after MMS exposure. MMS is a direct mono-methylating agent, which is known to methylate RNA nucleosides and DNA nucleosides. From our systematic study, we know that the main damage products of canonical nucleosides are m^1^A and m^7^G^10^. In the current study, we also focused on the quantities of ms^2^C formed during an LD_50_ MMS exposure of *E. coli*. We find 0.1 m^1^A damage sites per average tRNA and surprisingly 0.05 ms^2^C per average tRNA. At lower MMS doses (3 mM), the number of m^1^A damage sites is reduced to 0.014 per average tRNA and 0.018 ms^2^C per average tRNA. It is now clear that ms^2^C is an equally prominent damage product as m^1^A in total tRNA. If one considers that the average tRNA in bacteria is composed of 15 adenosines while only 0.1 s^2^C are found in the average tRNA, our results are rather intriguing. Although adenosine is 150 fold more abundant than s^2^C in total tRNA, the methylation products m^1^A and ms^2^C are of comparable quantities. From our data, we conclude that s^2^C is an at least equal or even better substrate of direct methylation than adenosine in the tRNA of the bacterium *E. coli*.

Although the abundance of modified nucleosides is low in comparison to the amount of canonical nucleosides in the total RNA pool, modified nucleosides fulfil a variety of important functions within the cell. The damage of a modified nucleoside will most likely interrupt the modification’s function and might be disadvantageous to the organism. We have tested how the methylation of s^2^C influences the translation in vivo. Serine can be translated by tRNA^Ser^_GGA_ (no s^2^C) reading the TCC codon or tRNA^Ser^_GCU_ reading the AGC or AGU codons. We found that *sfgfp* with the AGC or AGU codon was less efficiently translated than *sfgfp* with the TCC codon under MMS stress. This finding indicates that the translation of the AGC and AGT codon is disturbed after MMS stress, potentially due to the presence of ms^2^C.

The repair of damaged ribonucleosides might occur by two potential mechanisms. RNA is a transient molecule and constantly transcribed from DNA within the cell. Upon damage, it is possible to remove the damage by controlled degradation of the RNA. Due to the fact that tRNA maturation and modification is energy consuming, the second repair scenario of direct damage repair by, e.g., demethylation, is more attractive. In bacterial RNA, the repair of m^1^A and m^3^C by oxidative demethylation by the enzyme AlkB has been shown^7^. In our NAIL-MS studies, we use stable isotopes such as carbon-13, nitrogen-15 or sulfur-34 to distinguish RNA that was present during the stress event (here, MMS exposure) and new RNA transcribed during the recovery period. We can thus distinguish repair by RNA degradation and demethylation. With NAIL-MS, we observe AlkB dependent demethylation of ms^2^C, m^3^C and potentially m^1^A.

The AlkB dependent repair of m^3^C had been suggested in previous studies but has never been shown in vivo. Surprisingly, m^1^A repair was similar in the WT and Δ*alkB* mutant. Only for the 3 mM MMS dose, 3 h after stress exposure, the WT strain showed less m^1^A per tRNA compared to the Δ*alkB* strain. This is in accordance with the only other in vivo study, where m^1^A repair by AlkB was observed in radioactive tRNA 3 h after stress at a low dose of alkylating agent^23^.

In unstressed Δ*alkB* bacteria, we did not observe an accumulation of ms^2^C (Supplementary Fig. 21). This is to be expected if one considers the low copy number of AlkB in unstressed wildtype bacteria (1 copy of AlkB in log phase *E. coli* cells^25^). Only after exposure to alkylating agents, *alkB* is induced and six hours after 3 mM MMS exposure 6000 AlkB copies/cell have been found^11^.

Concerning ms^2^C repair, we found an increase of original [^32^S]-s^2^C in vivo, which reflects direct sulfur demethylation by AlkB. With our sophisticated NAIL-MS approach we could additionally observe an AlkB independent formation of s^2^C which originated from dethiomethylation of s^2^C to cytidine and subsequent rethiolation. The initial dethiolation step might be enzymatically catalyzed, but due to the electrophilic nature of the C2 in ms^2^C, a direct nucleophilic attack by water is also possible.

So far, m^1^A was thought to be the main methylation damage product in bacterial RNA and thus to be the main substrate of AlkB. Our data shows that ms^2^C is a methylation damage in bacterial tRNAs of comparable extent after MMS exposure. In addition, AlkB is faster during the repair of ms^2^C damaged tRNAs compared to m^1^A damaged tRNAs.

Since its discovery, AlkB has been an enzyme full of surprises. In this work, we present the next surprise, which is AlkBs extended substrate repertoire and its preference for the sulfur methylated nucleoside ms^2^C.

## Methods

### Salts, reagents, and nucleosides

All salts were obtained from Sigma-Aldrich (Munich, Germany) at molecular biology grade unless stated otherwise. The isotopically labeled compounds ^15^NH_4_Cl (>98 atom %) and [D_3_]-L-methionine (98 atom %) were obtained from Sigma-Aldrich. Isotopically labeled ^13^C_6_- glucose (≥99 atom %) and isotopically labeled Na_2_^34^SO_4_ (99.11 atom %) were obtained from Eurisotope (Saarbruecken, Germany). All solutions and buffers were made with water from a Millipore device (Milli-Q, Merck, Darmstadt, Germany). The nucleosides adenosine, cytidine, guanosine, uridine, and N2-methylguanosine (m^2^G) were obtained from Sigma-Aldrich. 1-Methyladenosine (m^1^A), 2-methyladenosine (m^2^A), N3-methylcytidine (m^3^C), N6-methyladenosine (m^6^A), 7-methylguanosine (m^7^G), 5-methylcytidine (m^5^C), 5-methyluridine (m^5^U), 2’-O-methylcytidine (Cm), 2′-O-methylguanosine (Gm), 1-methylguanosine (m^1^G), and 3-methyluridine (m^3^U) were obtained from Carbosynth (Newbury, UK).

### Synthesis of 2-methylthiocytidine

Twenty milligrams of s^2^C were stirred in 1 mL anhydrous ethanol under nitrogen atmosphere.7.4 mg of NaHCO_3_ and 24 µL of methyl iodide were added and the reaction was stirred overnight at room temperature. The clear yellow solution was evaporated and two times purified by silica gel chromatography in dichloromethane with 10 % methanol. The Rf value (TLC) in DCM/MeOH (10:1) was 0.46.

^1^H NMR (400 MHz, D_2_O):δ = 8.39 (d, J = 7.7 Hz, 1H, H-6), 6.60 (d, J = 7.7Hz, 1H, H-5), 5.98 (d, J = 3.0Hz, 1H, H-1′), 4.38 (t, J = 3.4 Hz, 1H, H-2′), 4.23-4.18 (m, 2H, H-3′, H-4′), 3.99 (d, J = 12.7Hz, 1H, H-5′a), 3.85 (dd, J = 2.7 Hz, 13.4 Hz, 1H, H-5′b), 2.67 (s, 3H, CH_3_). ^13^C NMR (500 MHz, D_2_O):δ = 166.4 (C-2), 162.1 (C-4), 141.5 (C-6), 102.1 (C-5), 92.7 (C-1′), 84.6 (C-3′), 74.9 (C-2′), 68.5 (C-4′), 59.8 (C-5′), 14.4 (C-7).

### *E. coli* and *P. aeruginosa* strains

The used *E. coli* wild-type strain BW25113 and the isogenic knockout strains were purchased from the Keio database^26^. *Pseudomonas aeruginosa* PA14 was a generous gift of Prof. Peter Dedon. Knockout strains with kanamycin resistance were grown on kanamycin LB agar plates (50 µg/mL). The *E. coli* strain AG1 (ME5305) transformed with the indicated pCA24N-based vectors was used for gene overexpression and ordered from the ASKA library database^27^. Strains with chloramphenicol resistance were grown in the presence of chloramphenicol (30 µg/mL). All cultures were grown in a shaking incubator at 37 °C at 250 rpm (Orbit = 10 mm). Overnight cultures were grown in 5 mL of the media used for the respective experiment. The cells were grown starting with an OD_600_ of 0.1, 0.5 or 1 (as specified in the respective section) and grown until reaching stationary phase (OD_600_ ~ 4). 

### Growth media LB/ M9/ isotope labeled

For LB media, LB Broth (Luria Miller) from Roth (Karlsruhe, Germany) was used. 1.5% (wt/vol) agar plates were prepared with LB-broth and Agar-Agar (Kobe I from Roth) according to manufacturer’s protocol.

M9 minimal medium was used with and without the indicated isotopes. Unlabeled M9 was prepared by mixing a 10 × M9 stock solution with glucose, MgCl_2_, Na_2_SO_4_, and CaCl_2_ (as detailed below). For unlabeled 10 × M9 stock solution, Na_2_HPO_4_ (68 g/L), KH_2_PO_4_ (30 g/L), NaCl (2.5 g/L), and NH_4_Cl (10 g/L) were mixed and autoclaved. For ^15^N-labeled 10 × M9 stock solution, ^15^NH_4_Cl (10 g/L) was used. MgCl_2_ (0.1 M), CaCl_2_ (0.1 M), Na_2_SO_4_ (0.1 M), and 20% (wt%) glucose were prepared by sterile filtration. A 20% (wt%) ^13^C_6_-labeled glucose solution was prepared for ^13^C-labeled M9 media. For ^34^S-labeled M9 media a 0.1 M Na_2_^34^SO_4_ solution was prepared. Final M9 media was prepared by mixing, e.g., 500 μL M9 stock solution with 100 μL glucose, 100 μL MgCl_2_, 100 μL Na_2_SO_4_, 5 μL CaCl_2_ and water to a final volume of 5 mL. For ^15^N-labeled cultures, the ^15^N-10 × M9 stock solution was used. For ^13^C-labeled cultures, the 20% (wt%) ^13^C_6_-labeled glucose solution and for ^34^S-labeled cultures, the 0.1 M Na_2_^34^SO_4_ solution was used. For CD_3_-methylome labeling, 200 μL CD_3_-methionine (stock 5 g/L) was added to 5 mL of culture volume.

### *E. coli* knockout/overexpression strain library

The knockout strains were cultured in LB or M9 media, starting from OD_600_ = 0.5. After 3 h the cells were harvested and the RNA was isolated and purified. The overexpression strains were cultured in LB media starting with an OD_600_ = 0.5 and an IPTG concentration of 0.1 mM. After 1 h incubation at 37 °C and 250 rpm (Orbit = 10 mm) the IPTG concentration was increased to 1 mM to induce full overexpression. The bacteria were incubated for further 2 h before the cells were harvested and RNA was isolated and purified

### Survival assay

Unlabeled M9 media was used throughout the survival assay. A 5 mL culture with OD_600_ 1.0 (or 0.1, respectively) were prepared from an *E. coli* overnight culture. After 60 min incubation (30 min with Streptozotocin), 100 µL of the culture were diluted 10^−5^ or 10^−6^ with sterile water. From this dilution 70 µL were plated on a prewarmed LB agar plate. The colony number of this plate represents 100% bacterial survival. MMS (Methyl-methanesulfonate, 99%) or Streptozotocin (STZ, 10 mM stock solution) was added to the remaining bacteria in defined concentrations. After 60 min (30 min) exposure, 100 µL of the culture were diluted and plated. The LB plates were incubated at 37 °C overnight and the colonies counted for determination of the survival at the respective MMS or STZ concentration.

### *E. coli* incubation with Streptozotocin (STZ)

From an overnight culture of *E. coli* strain BW25113 (WT) the OD_600_ was brought to 1.0 and the culture was grown for 1 h. To 5 mL bacterial culture 125 µL of a 10 mM STZ solution was added (200 µM final conc.) and the culture was incubated for 30 min. Afterwards, the bacteria were centrifuged and the resulting pellet was used for RNA isolation and purification.

### Cell lysis and tRNA purification

The bacteria culture was centrifuged at 1200 × *g* for 5 min. The supernatant was discarded and the cell pellet was resuspended in 1 mL TRI reagent (Sigma-Aldrich) per 5 mL bacteria culture. The total RNA was isolated according to the supplier’s manual. tRNA was purified by size exclusion chromatography (SEC) according to published procedures^10^. The tRNA was resuspended in water (30 μL).

### Isoacceptor purification

The procedure was adapted from Hauenschild et al.^22^. One microgram of total tRNA was incubated with 100 pmol of the biotinylated DNA probe and purified using Dynabeads T1 (Thermo Fisher Scientific, Waltham, MA, USA) according to the manusfacturer’s protocol. For tRNA isoacceptor purification, pre-purified total tRNA was used. The sequences of the biotinylated 2′-deoxyoligonucleotide probes are listed in Supplementary Table 4.

### Comparative NAIL-MS

One *E. coli* culture was grown overnight in an unlabeled M9 medium and another in a ^13^C labeled M9 medium. From these cultures, a labeled and unlabeled exposure culture with an OD_600_ of 1.0 and a volume of 5 mL were prepared. After 60 min growth, 8.5 μL MMS (final conc. 20 mM) were added to the unlabeled culture and 8.5 μL of water to the ^13^C-labeled culture (MOCK). After 60 min of exposure both cultures were mixed and the RNA was purified immediately. Total tRNA and tRNA isoacceptors were isolated as described above. For validation of the comparative NAIL-MS assay, the experiment was repeated by mixing and co-purifying RNA (total tRNA and tRNA isoacceptors) from an unlabeled MOCK treated culture and a ^13^C-labeled MOCK treated culture.

### Methylome discrimination assay

For this assay, CD_3_-labeled M9 medium was used at all times. A 5 mL bacterial solution with an OD_600_ of 0.1 was prepared from an overnight culture. After 60 min growth, 8.5 μL MMS (final conc. 20 mM) were added. As a control, 8.5 µL water was added to a second culture. After 60 min of exposure, the RNA was isolated and total tRNA purified by SEC.

### sfGFP reporter and single-cell fluorescence microscopy

For in vivo quantification of the effects of tRNA modifications after MMS stress on translation, the *E. coli* strains BW25113, BW25113 Δ*alkB* and BW25113 Δ*ttcA* were transformed with pBAD24 containing a copy of sf*gfp* under the control of the arabinose-inducible P_BAD_ promoter. To test the effects of different serine codons (AGC, AGT, TCC) the only serine codon of the used sf*gfp* sequence (corresponds to amino acid position 2) was exchanged by using the respective primers during the cloning process. To enhance the number of serines, the sf*gfp* sequence was extended at the 5′-end with multiple motifs coding for proline-alanine-serine (ASPAAPSASAPSAASAAPSAA)-sequence, modified from previous studies^28^. We used the primers PAS-gfp_for/ PAS-AGT_gfp_for / PAS-TCC_gfp_for plus gfp_rev to introduce different types of codons for serine (AGC, AGT or TCC) in the sfGFP variants extended with the proline-alanine-serine (ASPAAPSASAPSAASAAPSAA)-sequence or respectively gfp_for / AGT_gfp_for / TCC_gfp_for plus gfp_rev to produce the sfGFP variants with different types of codons for serine (AGC, AGT or TCC). The restrictions sites EcoRI and XbaI were used for integration of the sf*gfp* variants into the pBAD24 plasmid.

For each experiment cells of an overnight culture were inoculated into fresh M9 minimal medium supplemented with 0.5 % (vol/vol) glycerol as a sole carbon source and incubated under vigorous shaking at 37 °C. Cells were grown to mid-log phase, and the culture was divided. One half was stressed by the addition of MMS to final concentrations of 3 and 20 mM, respectively, the other half remained untreated. After 1 h of incubation in presence of MMS the medium was exchanged with fresh, pre-tempered M9 minimal media supplemented with 0.5% (vol/vol) glycerol and 0.2% (vol/vol) arabinose to induce sfGFP expression. As a control, cells were cultivated without addition of arabinose. To measure sfGFP fluorescence, cells were fixed on an agarose pad (1% wt/vol in phosphate-buffered saline) placed on a microscope slide with coverslip. Micrographs were taken on a Leica microscope DMI 6000B equipped with a Leica DFC 365Fx camera (Andor, 12 bit). sfGFP fluorescence was visualized using an excitation wavelength of 460 nm and a 512 nm emission filter with a 75-nm bandwidth. Fluorescence intensities of a minimum of 300 cells per transformant were collected and quantified using Fiji^29^.

### Pulse-chase NAIL-MS experiment

A single colony of *E. coli* BW25113 or *E. coli* JW2200-KC (Δ*alkB*) was picked and grown in unlabeled M9 medium (5 mL) overnight. From the first overnight culture, a 50 mL culture was prepared in unlabeled M9 medium and grown overnight. From the second overnight culture, 120 mL culture (OD_600_ of 1.0) was prepared in unlabeled M9 medium. After 60 min growth, the first aliquot (7 mL) was taken for RNA isolation. The remaining culture was split into two flasks of 56.5 mL each. One was exposed to MMS (95.7 μL, 20 mM final concentration) the other to water (MOCK) and inverted before both cultures were cultivated for 60 min. An aliquot (7 mL) was drawn from each culture, and the RNA was isolated. The remaining bacteria were centrifuged (1200 × *g*, 5 min), and the MMS/MOCK-containing supernatants were discarded. The bacteria pellets were washed with ^15^N, ^34^S and CD_3_-methionine labeled M9 medium (5 mL), and each bacterial pellet was suspended in fresh ^15^N/^34^S/CD_3_ M9 medium (50 mL). The bacteria were allowed to grow and recover from the MMS/MOCK treatment. Seven millilitres of each bacterial culture were harvested after 1, 2, 3, 4, and 23 h. The RNA was isolated and the tRNA purified by SEC. The experiment was also done with lower volumes and different concentrations of MMS.

### SAM and MMS incubation assay in vitro

4.3 μL of a 93 μM s^2^C synthetic standard solution (0.4 nmol) were mixed with 0, 500, 1000, 1500 or 2000 equivalents of SAM (32 mM stock) or MMS (20 mM stock) in a final volume of 100 μL containing 50 mM Na_2_HPO_4_/NaH_2_PO_4_ buffer (pH 7.0). The mixture was incubated at 37 °C for 60 min in a shaking heat block. After incubation, 900 µL water were added (1:10 dilution) and spiked with SILIS to be analyzed by quantitative LC-MS/MS.

### AlkB in vitro assay

Five microlitres of purified tRNA (30 ng/µL) from 20 mM MMS exposed *E. coli* was mixed with 45 μL of AlkB. (45 µL AlkB was prepared with 7.5 μL KCl (100 mM), 1.5 μL freshly mixed sodium α-ketoglutarate (10 mM), 2.5 μL Tris buffer (1 M, pH 7.65), 5 μL freshly mixed sodium L-ascorbate (20 mM), 1.5 μL freshly mixed Fe(II)(NH_4_)_2_(SO_4_)_2_ (10 mM) and 1.5 μL of AlkB enzyme (39.1 μM, Peak Proteins, Cheshire, UK) and water.) The reaction mixture was incubated at 37 °C for 60 min. After incubation, 500 μL of LiClO_4_ in acetone (2 vol%) was added for tRNA precipitation and mixed thoroughly. After 10 min at room temperature, the sample was centrifuged at 5000 × *g* for 10 min. The RNA pellet was washed with 70 vol% ethanol and resuspended in 20 µL water before digestion for LC-MS/MS analysis.

### tRNA digestion for mass spectrometry

tRNA (100 ng) in aqueous digestion mix (30 μL) was digested to single nucleosides by using 0.2 U alkaline phosphatase, 0.02 U phosphodiesterase I (VWR, Radnor, Pennsylvania, USA), and 0.2 U benzonase in Tris (pH 8, 5 mM) and MgCl_2_ (1 mM) containing buffer. Furthermore, 0.5 µg tetrahydrouridine (Merck, Darmstadt, Germany), 1 µM butylated hydroxytoluene, and 0.1 µg pentostatin were added to avoid deamination and oxidation of the nucleosides^30^. The mixture was incubated for 2 h at 37 °C and then filtered through 96-well filter plates (AcroPrep Advance 350 10 K Omega, PALL Corporation, New York, USA) at 3000 × *g* and 4 °C for 30 min. 1/10 Vol. of SILIS (stable isotope labeled internal standard) as prepared in^31^ was added to each filtrate before analysis by QQQ mass spectrometry.

### High resolution mass spectrometry

The ribonucleosides were separated using a Dionex Ultimate 3000 HPLC system on an Interchim Uptisphere120-3HDO C18 or a Synergi, 2.5 μm Fusion-RP C_18_, 100 Å, 100 × 2 mm (Phenomenex®, Torrance, California, USA). Mobile phase A was 2 mM ammonium acetate and mobile phase B was 80% acetonitrile containing 2 mM ammonium acetate. Gradient elution started with 0% B and increased to 12% B after 10 min and to 80% after 12 min. After 4 min elution at 80% B and subsequently regeneration of starting conditions to 100% A after 5 min, the column was equilibrated at 100% A for 8 min. The flow rate was 0.2 mL/min and the column temperature 30 °C. High-resolution mass spectra of precursor and product ions were recorded by a ThermoFinnigan LTQ Orbitrap XL. The parameters of the mass spectrometer were tuned with a freshly mixed solution of adenosine (5 μM). The parameters were sheath gas flow rate, 16 arb; auxiliary gas flow rate, 11 arb; sweep gas flow rate, 4 arb; spray voltage, 5.0 kV; capillary temperature, 200 °C; capillary voltage, 20 V, tube lens 65 V.

### QQQ mass spectrometry

For quantitative mass spectrometry an Agilent 1290 Infinity II equipped with a diode-array detector (DAD) combined with an Agilent Technologies G6470A Triple Quad system and electrospray ionization (ESI-MS, Agilent Jetstream) was used. Operating parameters: positive-ion mode, skimmer voltage of 15 V, cell accelerator voltage of 5 V, N_2_ gas temperature of 230 °C and N_2_ gas flow of 6 L/min, sheath gas (N_2_) temperature of 400 °C with a flow of 12 L/min, capillary voltage of 2500 V, nozzle voltage of 0 V, and nebulizer at 40 psi. The instrument was operated in dynamic MRM mode.

For separation a Core-Shell Technology column (Phenomenex, Torrance, CA, USA; Kinetex 1.7 μm EVO C_18_, 100 Å, 150 × 2.1 mm) at 35 °C and a flow rate of 0.35 mL/min were used in combination with a binary mobile phase of 5 mM NH_4_OAc aqueous buffer A, brought to pH 5.6 with glacial acetic acid (65 μL), and an organic buffer B of pure acetonitrile (Roth, LC-MS grade, purity ≥.99.95). The gradient started at 100% solvent A, followed by an increase to 10% over 10 min. From 10 to 15 min, solvent B was increased to 45% and was maintained for 3 min before returning to 10% solvent A and a 3 min re-equilibration period.

### Calibration

For calibration, synthetic nucleosides were weighed and dissolved in water to a stock concentration of 1–10 mM. Due to an unknown content of water and salts in the synthesized ms^2^C standard, the concentration could not be determined through weighing. Therefore, the concentration of the stock solution was determined by comparison to s^2^C containing isoacceptors after MMS exposure. The calibration solutions range from 0.3 to 500 pmol for each canonical nucleoside and from 0.3 to 500 fmol for each modified nucleoside and were spiked with 10% SILIS^20^. The sample data were analyzed by the Quantitative and Qualitative MassHunter Software from Agilent. The areas of the MRM signals were integrated for each modification and their isotope derivatives.

The absolute amounts of the modifications were referenced to the absolute amounts of the respective canonical. In the case of the pulse-chase experiment, the different isotopomers were referenced to their respective labeled canonicals, so that original modifications were referenced to original canonicals and new modifications were referenced to new canonicals. See Eqs. (1) and (2) for s^2^C as an example in Supplementary Table 12.

### Statistics

All experiments were performed at least three times (biological replicates) to allow student *t*-test analysis. *p*-values of student *t*-test (unpaired, two-tailed, equal distribution) were calculated using Excel or Graphpad Prism.

### Reporting summary

Further information on research design is available in the Nature Research Reporting Summary linked to this article.

## Supplementary information

Supplementary Information

Peer Review

Reporting Summary

## Data Availability

A reporting summary for this Article is available as a Supplementary Information file. The source data underlying Figs. 1c, d, 2a–c, 3a, c, 4b–e, 5b, c and Supplementary Figs. 2a, 5–8, 10–18, 20, 21 as well as Supplementary Table 10 are provided as a Source Data file. All relevant data are available from the corresponding author upon reasonable request.

## Source data

Source Data
