# Supplementary material for: NAIL-MS reveals the repair of 2-methylthiocytidine by AlkB in E. coli
Source: Nat Commun. 2019 Dec 6;10:5600. doi: 10.1038/s41467-019-13565-9 (PMC6898146; doi:10.1038/s41467-019-13565-9)
Supplement: Supplementary file 1 — Supplementary Information [file 41467_2019_13565_MOESM1_ESM.pdf]

Supplement

NAIL-MS reveals the repair of 2-methylthiocytidine by AlkB in *E. coli*

Valentin F. Reichle[1], Dimitar P. Petrov[2], Verena Weber[1], Kirsten Jung[2] and Stefanie Kellner[1]*

[1] Department of Chemistry, Ludwig-Maximilians-University Munich, Butenandtstr. 5-13, 81377 Munich, Germany

[2] Department of Biology, Ludwig-Maximilians-University Munich, Grosshaderner Str. 2-4, 82152 Martinsried, Germany

* Corresponding author: stefanie.kellner@cup.lmu.de

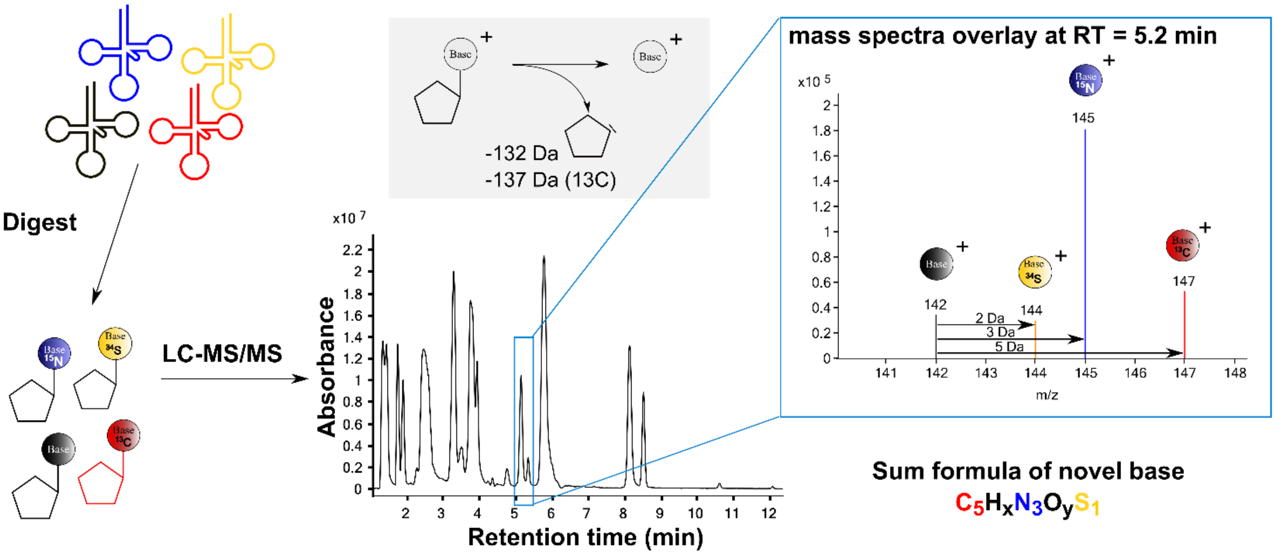

Supplementary Figure 1: Principle concept for the discovery of unknown nucleosides. Bacteria (*E. coli* or *P. aeruginosa*) are fed with stable isotope labeled nutrients in minimal M9 medium supplemented with glucose. The completely carbon-13 (red), nitrogen-15 (blue), sulfur-34 (yellow) or unlabeled (black) tRNAs are isolated and digested to nucleosides. Each RNA digest of isotopically labeled bacteria is analyzed by LC-MS/MS and the total ion count is displayed in the chromatogram. The mass spectra of the chromatographic peaks are isolated and overlayed for the differentially labeled samples. Here, the mass spectra overlay for the new compound ms^2C at retention time (RT) 5.2 min is shown.

Supplementary Table 1a: Parameters of Triple Quadrupole method used for the discovery of ms^2C. The difference between precursor and product ions is 132 units, the common fragmentation of nucleosides (loss of ribose). The table applies only for unlabeled, nitrogen-15 and sulfur-34 labeled RNA samples.

Precursor Ion	Product Ion	Ret. Time	Delta Ret.	Fragmentor	Collision energy	Cell Accelerator Voltage	Polarity
240	108	10	20	250	40	2	positive
241	109	10	20	250	40	2	positive
242	110	10	20	250	40	2	positive
243	111	10	20	250	40	2	positive
..	..	10	20	250	40	2	positive
..	..	10	20	250	40	2	positive
419	287	10	20	250	40	2	positive
420	288	10	20	250	40	2	positive

Supplementary Table 1b: Parameters of Triple Quadrupole method used for the discovery of ms^2C. The difference between precursor and product ions is 137 units, which reflects the loss of a $^{13}C_5$-labeled ribose.

Precursor Ion	Product Ion	Ret. Time	Delta Ret.	Fragmentor	Collision energy	Cell Accelerator Voltage	Polarity
240	103	10	20	250	40	2	positive
241	104	10	20	250	40	2	positive
242	105	10	20	250	40	2	positive
243	106	10	20	250	40	2	positive
..	..	10	20	250	40	2	positive
..	..	10	20	250	40	2	positive
419	282	10	20	250	40	2	positive
420	283	10	20	250	40	2	positive

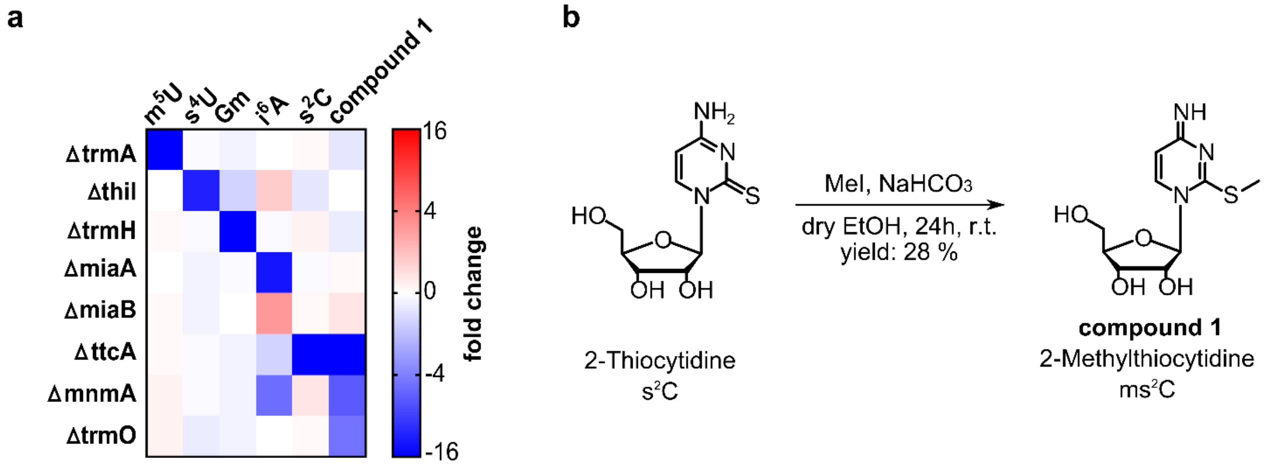

Supplementary Figure 2: Heatmap of *E. coli* knockout screen and synthetic route of ms^{2}C**. a.** tRNA from the indicated *E. coli* knockout strains was analyzed for the abundance of modified nucleosides. The found abundance is related to the abundance found in wildtype BW25113 strain. Compound 1 is ms^{2}C. Blue: Lower abundance compared to wildtype. Red: Higher abundance compared to the wildtype. **b.** Synthetic pathway and conditions of the synthesis from s^{2}C to ms^{2}C under usage of methyl iodide.

Supplementary Table 2: *E. coli* knockouts used for RNA modification screening in **Supplementary Figure 2** and **6**. Reference: Keio collection.[1]

Keio code number	knockout gene	Enzyme	Mod. suppression
BW25113	-	-	-
JW3937-KC	trmA	TrmA	m^{5}U
JW0413-KC	thiI	ThiI	s^{4}U
JW3626-KC	trmH	TrmH	Gm
JW4129-KC	miaA	MiaA	i^{6}A
JW0658-KC	miaB	MiaB	ms^{2}i^{6}A
JW1338-KC	ttcA/ydaO	TtcA	s^{2}C
JW1119-KC	mnmA	MnmA	s^{2}U
JW0191-KC	trmO/yaeB	TrmO	m^{6}t^{6}A
JW2501-KC	yfgB	RlmN	m^{2}A
JW2762-KC	yqcB	TruC	Psi
JW0396-KC	tgt	TGT	Q
JW2459-KC	tmcA/ypfI	TmcA	ac^{4}C
JW2559-KC	trmN6/yfiC	TrmN6	m^{6}A
JW3228-KC	dusB	DusB	D

Supplementary Table 3: MRM parameters for LC-MS/MS analysis of unlabeled tRNA digests (*E.g.* for isoacceptor purification analysis or knockout library analysis of.

Compound Group	Compound Name	Precursor Ion (m/z)	Product Ion (m/z)	Ret Time (min)	Delta Ret Time (min)	Fragmentor (V)	Collision Energy (eV)	Cell Accelerator Voltage (V)	Polarity
unlabeled	C	244	112	1.73	1	175	13	5	Positive
	U	245	113	2	1	95	5	5	Positive
	G	284	152	3.718	3	95	17	5	Positive
	A	268	136	5.711	1	110	21	5	Positive
	s2C	260	128	2.5	2	85	13	5	Positive
	ms2C	274	142	5.533	3	85	13	5	Positive
	s4U	261	129	4.753	1	75	17	5	Positive
	Gm	298	152	5.133	1	100	9	5	Positive
	Cm	258	112	3.347	3	180	9	5	Positive
	I	269	137	3.556	1	100	9	5	Positive
	m2A	282	150	8.08	1	125	21	5	Positive
	m1G	298	166	5.08	1	105	13	5	Positive
	t6A	413	281	7.19	1	130	9	5	Positive
	m7G	298	166	2.021	2	105	14	5	Positive
	acp3U	346	214	1.744	2	180	20	5	Positive
	m5U	259	127	3.915	1	145	10	5	Positive
	Psi	245	209	1.49	1	90	5	5	Positive
	i6A	336	204	14.134	1	140	17	5	Positive
SILIS	C SILIS	256	119	1.73	1	175	13	5	Positive
	U SILIS	256	119	2	1	95	5	5	Positive
	G SILIS	299	162	3.718	3	95	17	5	Positive
	A SILIS	283	146	5.711	1	110	21	5	Positive
	s2C SILIS	272	135	2.5	2	85	13	5	Positive
	ms2C SILIS	287	150	5.533	3	85	13	5	Positive
	s4U SILIS	272	135	4.753	1	75	17	5	Positive
	Gm SILIS	314	162	5.133	1	100	9	5	Positive
	Cm SILIS	271	119	3.347	3	180	9	5	Positive
	I SILIS	283	146	3.556	1	100	9	5	Positive
	m2A SILIS	298	161	8.08	1	125	21	5	Positive
	m1G SILIS	314	177	5.08	1	105	13	5	Positive
	t6A SILIS	434	297	7.19	1	130	9	5	Positive
	m7G SILIS	314	177	2.021	2	105	14	5	Positive
	acp3U SILIS	362	225	1.744	2	180	20	5	Positive
	m5U SILIS	271	134	3.915	1	145	10	5	Positive
	Psi SILIS	256	220	1.49	1	90	5	5	Positive
	i6A SILIS	356	219	14.134	1	140	17	5	Positive

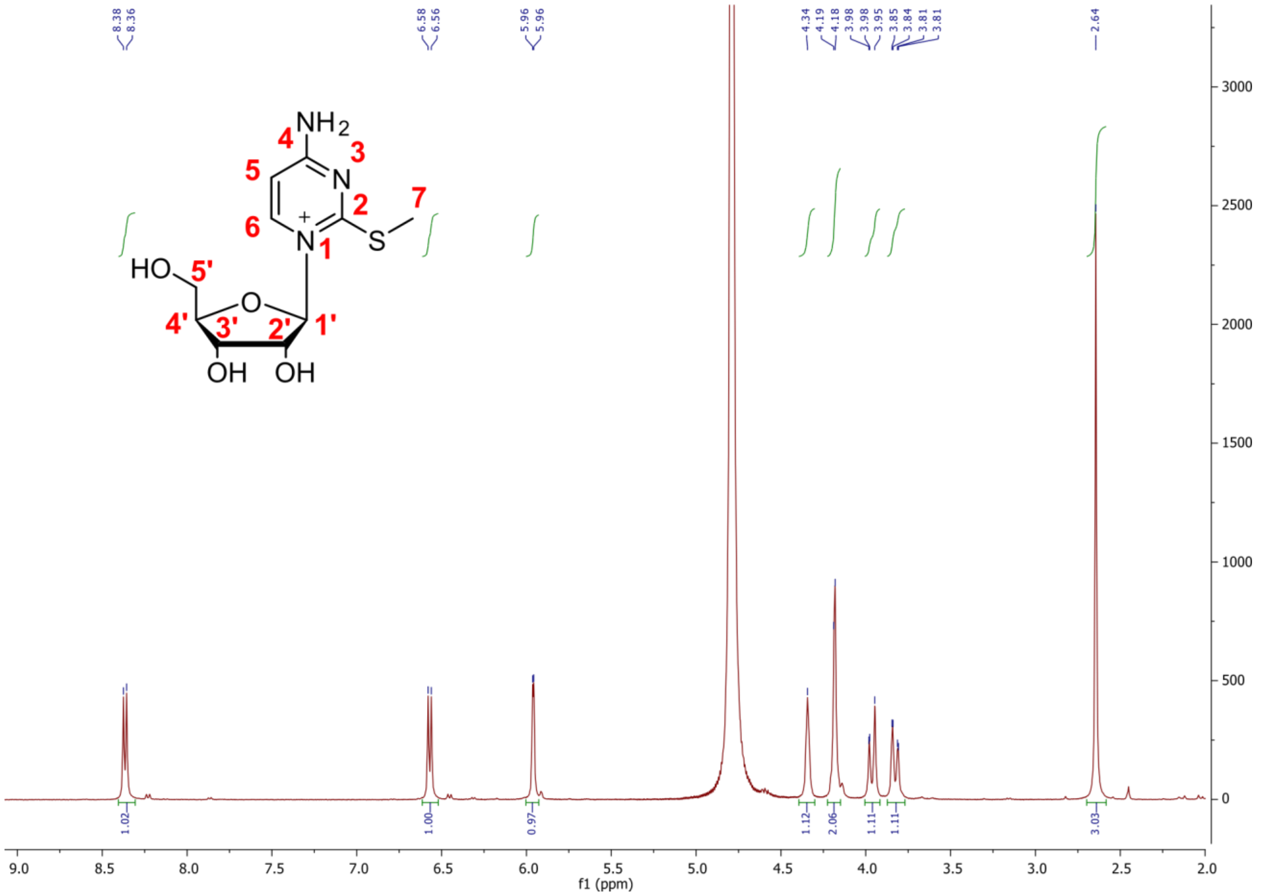

Supplementary Figure 3: 400 MHz ^{1}H-NMR spectrum of 2-methylthiocytidine in D_2O.

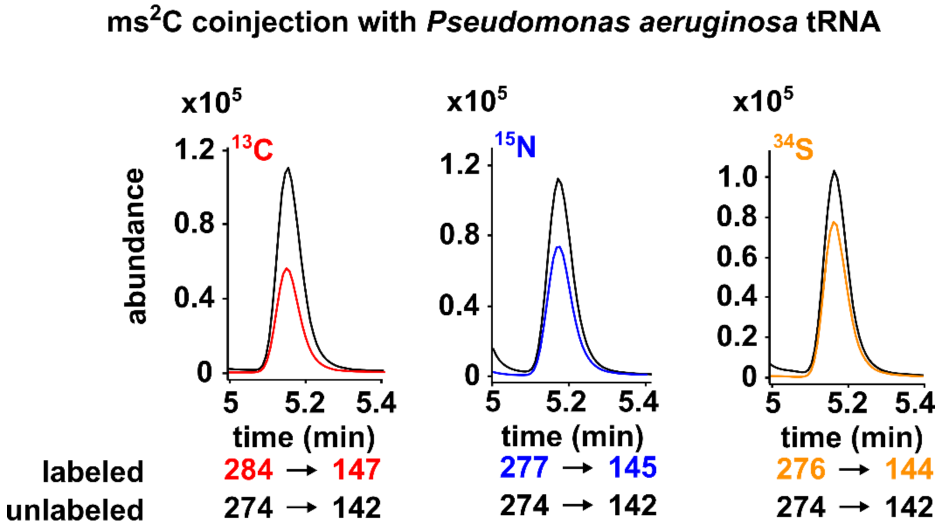

Supplementary Figure 4: Co-injection of synthetic ms^{2}C with digested and isotope labeled *P. aeruginosa* tRNA (grown in the indicated isotope labeled M9 medium). ms^{2}C was co-injected with fully ^{13}C labeled tRNA digest (red), with fully ^{15}N labeled tRNA digest (blue) and with ^{34}S labeled digest (orange). The mass transitions are shown below each chromatogram.

Supplementary Table 4: DNA oligonucleotides for tRNA isoacceptor purification. Every sequence starts with a Biotin [Btn]-tag, followed by three adenosines as a spacer before the actual reverse complementary sequence starts.

tRNA isoacceptor	Sequence of the probe (5‘-3‘)
$tRNA^{Arg}_{CCG}$:	[Btn]AAAGAGACCTCTGCCTCCGGAGGGCAGCGCTCTATCCAGCTGAGCTA
$tRNA^{Arg}_{ICG}$:	[Btn]AAACCGACCGCTCGGTTCGTAGCCGAGTACTCTACCAGCTGAGCTAC
$tRNA^{Arg}_{UCU}$:	[Btn]AAAGCGGCCCACGACTTAGAAGGTCGTTGCTCTATCCAACTGAGCTA
$tRNA^{Ser}_{GCU}$:	[Btn]AAACCCGGATGCAGCTTTTGACCGCATACTCCCTTAGCAGGGGAGC
$tRNA^{Ser}_{GGA}$:	[Btn]AAACCCCGATACGTTGCCGTATACACACTTTCCAGGCGTGCTCCTT
$tRNA^{Ser}_{UGA}$:	[Btn]AAATGGCGGAAGCGCAGAGATTCGAACTCTGGAACCCTTTCGGGTCGCCGGTTTCAAG
$tRNA^{Ala}_{UGC}$:	[Btn]AAATGGTGGAGCTATGCGGGATCGAACCGCAGACCTCCTGCGTGCA
$tRNA^{Gly}_{CCC}$:	[Btn]AAATGGAGCGGGCGAAGGGAATCGAACCCTCGTATAGAGCTTGGGAAGCTCTCGTTCTACCGAACTACGCCCGC
$tRNA^{Lys}_{mnm5s2UUU}$:	[Btn]AAATGGTGGGTCGTGCAGGATTCGAACCTGCGACCAATTGATTAAA

Supplementary Table 5: Modified nucleosides and their positions in the respective tRNA sequence of each isoacceptor related to this work. Reference: MODOMICS [2]

	Modifications and their positions in the respective tRNA sequence																
**Modification/ Isoacceptor**	**s^4U**	**Gm**	**D**	**s^2C**	**Cm**	**Inosine**	**mnm^5U**	**m^1G**	**mnm^5s^2U**	**cmo^5U**	**m^2A**	**ms^2i^6A**	**m^7G**	**acp^3U**	**ct^6A**	**m^5U**	**Psi**
**Arg CCG**	x	x	17, 20A	32	x	x	x	37	x	x	x	x	46	x	x	54	55
**Arg ICG**	8	x	17, 20A	32	x	34	x	x	x	x	37	x	46	47	x	54	55
**Arg UCU**	x	x	x	32	x	x	34	x	x	x	x	x	x	x	37	54	40, 55
**Ser GCU**	8	x	20	32	x	x	x	x	x	x	x	x	x	x	37	54	55
**Ser GGA**	8	18	20, 20A	x	x	x	x	x	x	x	x	x	x	x	x	54	40, 55
**Ser UGA**	8	18	20, 20A	x	32	x	x	x	x	34	x	37	x	x	x	54	55
**Ala UGC**	8	x	17	x	x	x	x	x	x	34	x	x	46	x	x	54	55
**Gly CCC**	8	x	20	x	x	x	x	x	x	x	x	x	x	x	x	54	55
**Lys UUU**	x	x	16, 17, 20	x	x	x	x	x	34	x	x	x	46	47	37	54	39, 55

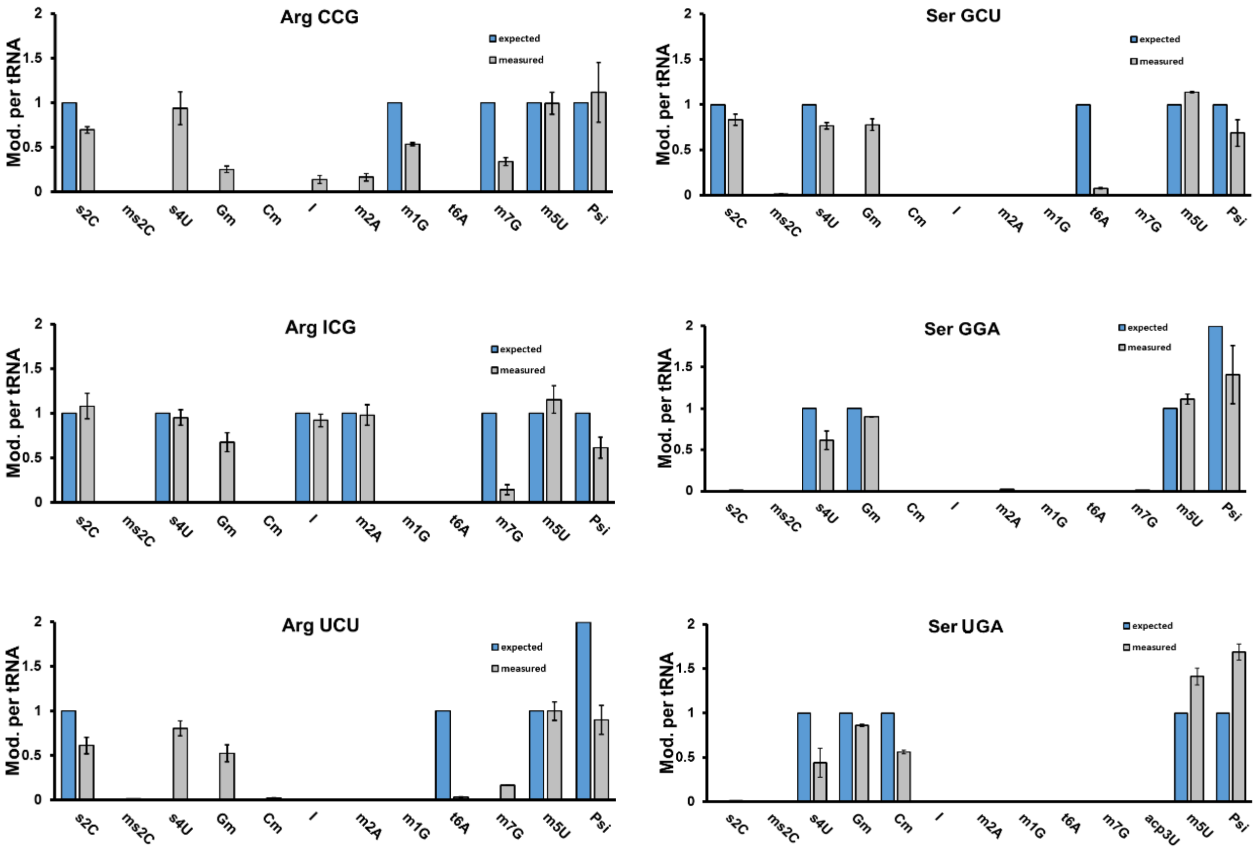

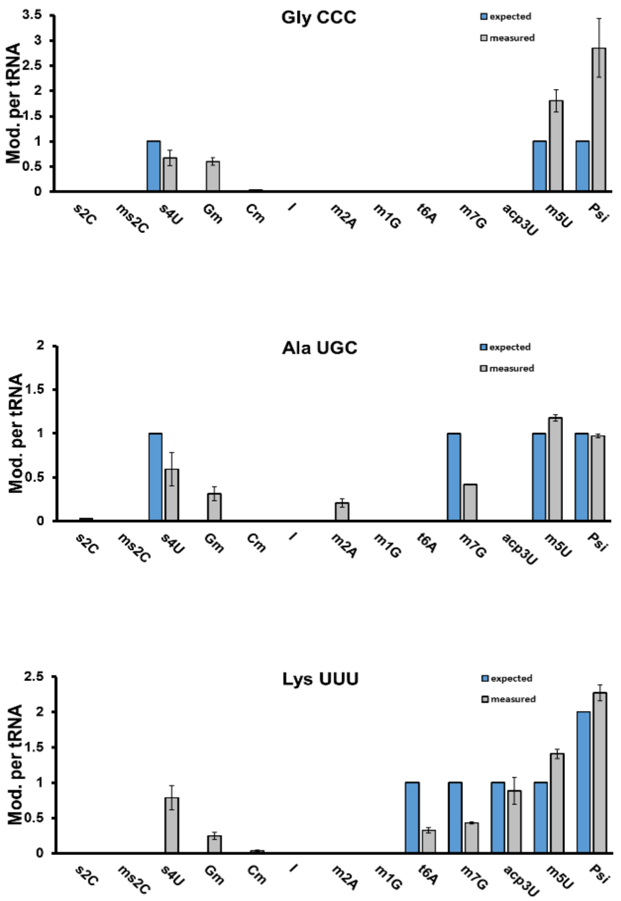

Supplementary Figure 5: Validation of tRNA isoacceptor purification by modified nucleoside analysis. Expected (blue, from MODOMICS) [2] and experimentally determined abundance of modified nucleosides per tRNA. *E.coli* was grown in LB medium. From n=3 biol. replicates, error bars reflect standard deviation. Source data are provided as a Source Data file.

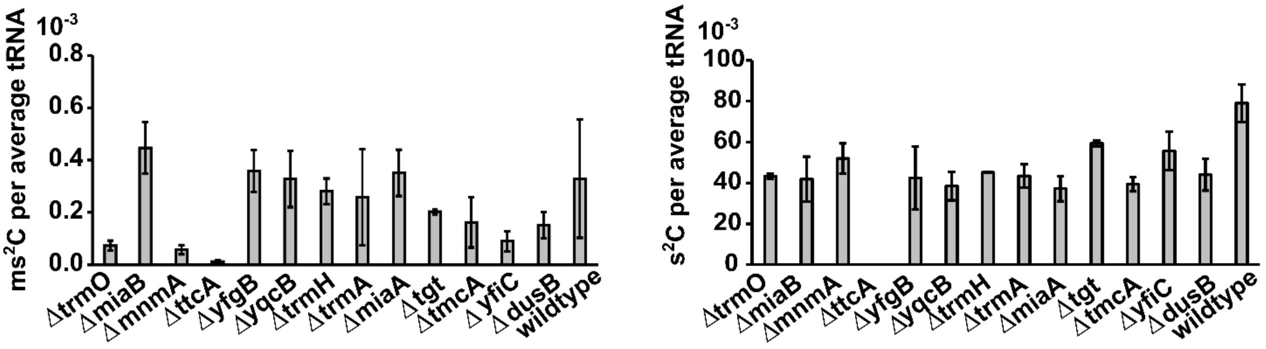

Supplementary Figure 6: *E. coli* knockout studies in M9 medium **Left:** ms^{2}C abundance in different *E. coli* RNA modification knockouts during stationary growth phase in M9 media. **Right:** s^{2}C abundance in different *E. coli* RNA modification knockouts during stationary growth phase in M9 media. All experiments are from n=3 biol. replicates and error bars reflect standard deviation. Source data are provided as a Source Data file.

Supplementary Table 6: *E. coli* overexpression strains used for RNA modification screening in **Supplementary Figure 7**. Reference: ASKA library [3]

ASKA code number	overexpressed	Enzyme	Modification
AG1 (ME5305)	-	-	-
AG1 (pCA24N-gfp)	-	empty vector	-
JW3228-AP	dusB	DusB	D
JW0396-AP	tgt	Tgt	Q
JW1119-AP	mnmA	MnmA	s^{2}C
JW0191-AP	tsaA/yaeB	TrmO	m^{6}t^{6}A
JW2559-AP	trmN6/yfiC	TrmN6	m^{6}A

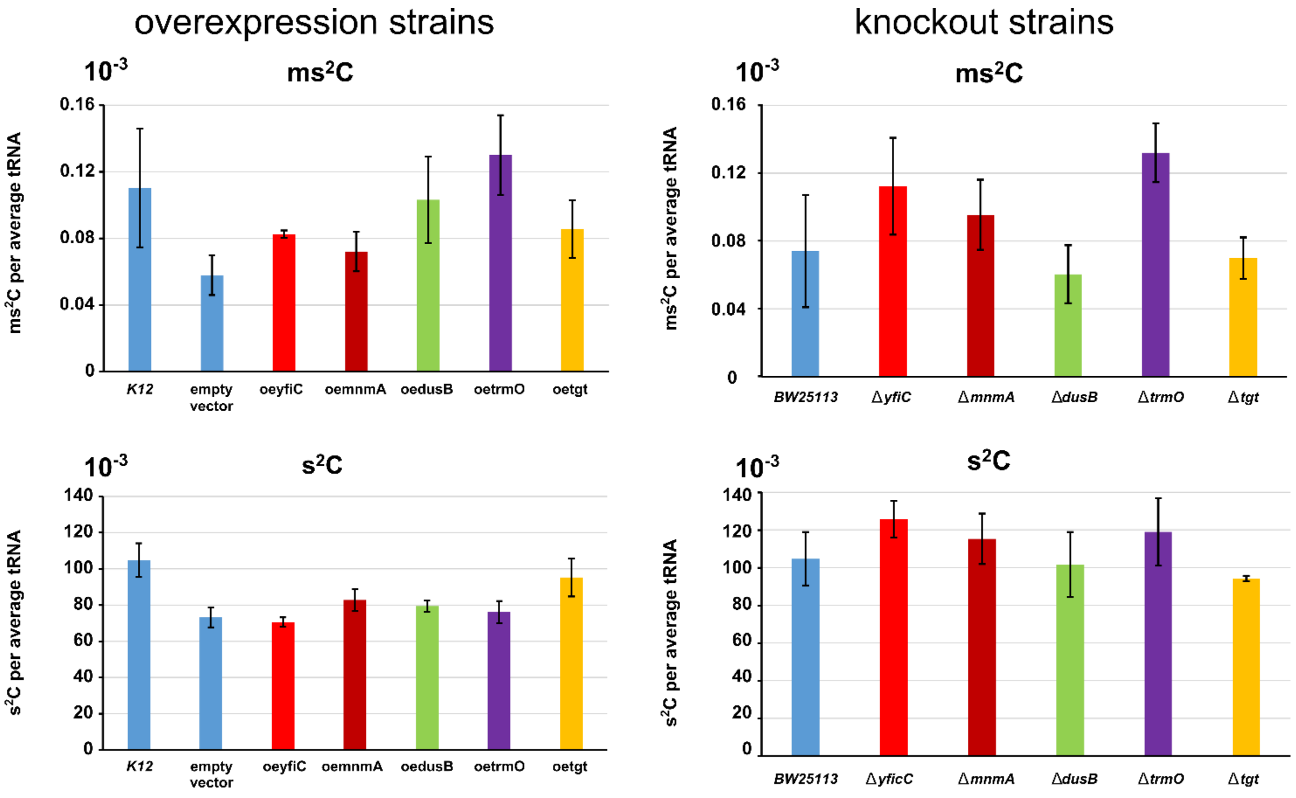

Supplementary Figure 7: ms^{2}C and s^{2}C abundance of *E. coli* knockouts and corresponding overexpression strains which showed low ms^{2}C abundance in M9 media before (Supplementary Figure 6). Here, they were cultured in LB media and harvested in exponential growth phase. As a control, the wildtype strains and the wildtype with the empty overexpressing gene vector are shown as well. The assumption of enzyme dependent ms^{2}C formation could not be confirmed. All experiments are from n=3 biol. replicates and error bars reflect standard deviation. Source data are provided as a Source Data file.

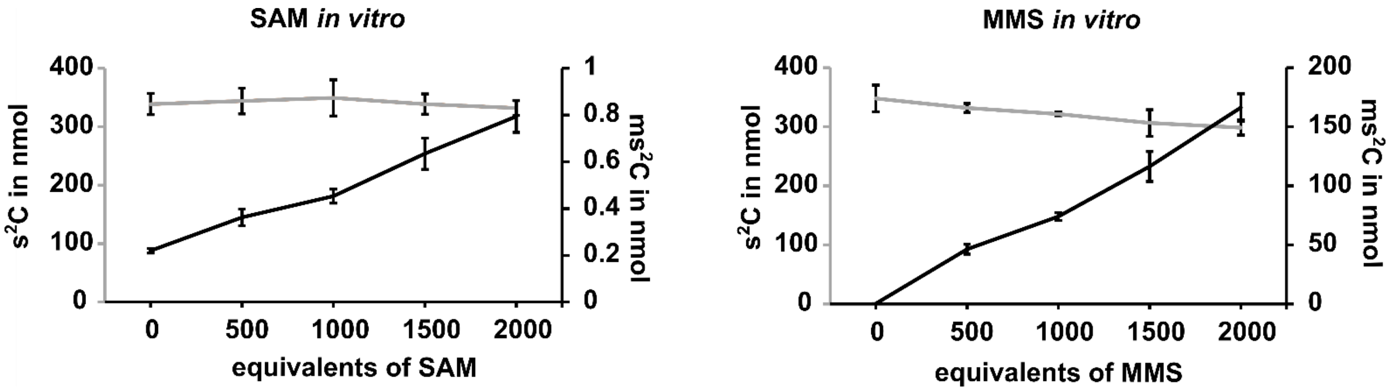

Supplementary Figure 8: *in vitro* incubation assay of s^{2}C with SAM and MMS. SAM: S-adenosylmethionine. MMS: Methyl-methanesulfonate. Grey axes and curve: abundance s^{2}C. Black axes and curve: abundance ms^{2}C. The experiments were done in triplicates and the error bars reflect standard deviation. Source data are provided as a Source Data file.

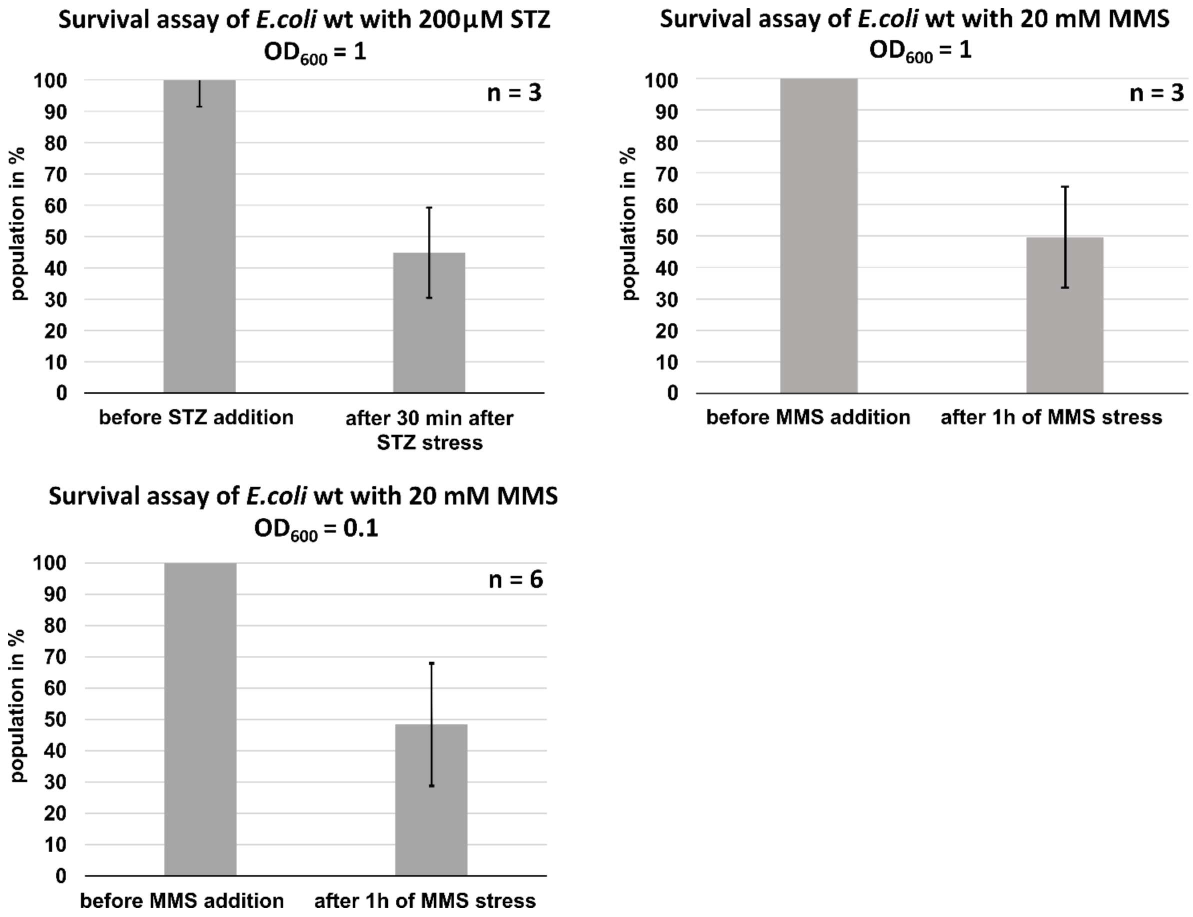

Supplementary Figure 9: Survival assay to determine the LD_{50} of MMS and Streptozotocin (STZ) for the *E.coli* strain BW25113. Cells were brought to either OD_{600} = 1 (top graphs, error bars reflect the standard deviation of n=3 biol. replicates) or OD_{600} = 0.1 (left bottom graph, error bars reflect the standard deviation of n=6 biol. replicates) and exposed to 200 µM STZ for 30 min. (left top graph) or 20 mM MMS for 60 min. (right top graph and left bottom graph).

Supplementary Table 7: MRM parameters for RNA methylome discrimination assay.

Compound Group	Compound Name	Precursor Ion (m/z)	Product Ion (m/z)	Ret Time (min)	Delta Ret Time (min)	Fragmentor (V)	Collision Energy (eV)	Cell Accelerator Voltage (V)	Polarity
unlabeled	C	244	112	1.73	1	380	40	5	Positive
	U	245	113	1.876	1	380	40	5	Positive
	G	284	152	3.718	3	380	40	5	Positive
	A	268	136	5.711	1	380	40	5	Positive
	ms2C	274	142	5.533	3	380	40	5	Positive
	m1A	282	150	1.745	2	380	10	5	Positive
	m3C	258	126	1.752	1	380	10	5	Positive
	i6A	336	204	14.134	1	380	10	5	Positive
	m7G	298	166	2.021	2	380	10	5	Positive
	m6A	282	150	8.423	1	380	10	5	Positive
	m2G	298	166	5.491	2	380	10	5	Positive
	m3U	259	127	4.696	2	380	10	5	Positive
	m5C	258	126	2.702	2	380	10	5	Positive
	m2A	282	150	8.08	1	380	10	5	Positive
	Gm	298	152	5.133	1	380	10	5	Positive
	m1G	298	166	5.08	1	380	10	5	Positive
	m5U	259	127	3.915	1	380	10	5	Positive
	ac4c	286	154	5.538	2	380	10	5	Positive
	cmnm5U	332	200	1.586	1	380	10	5	Positive
	I	269	137	3.556	1	380	10	5	Positive
	Cm	258	112	3.347	3	380	10	5	Positive
	m5s2U	275	143	6.172	2	380	10	5	Positive
	Am	282	136	7.257	1	380	10	5	Positive
	Psi	245	209	1.49	1	380	10	5	Positive
	s4U	261	129	4.753	1	380	10	5	Positive
	Um	259	113	4.49	1	380	10	5	Positive
	ms2i6A	382	250	10	20	380	10	5	Positive
	s2C	260	128	2.288	1	380	10	5	Positive
	t6A	413	281	7.553	1	380	10	5	Positive
	mnm5U	288	156	1.491	1	380	10	5	Positive
CD3 labeled	ms2C CD3	277	145	5.533	3	380	40	5	Positive
	m1A CD3	285	153	1.745	2	380	10	5	Positive
	m3C CD3	261	129	1.752	1	380	10	5	Positive
	m7G CD3	301	169	2.021	2	380	10	5	Positive
	m6A CD3	285	153	8.423	1	380	10	5	Positive
	m2G CD3	301	169	5.491	2	380	10	5	Positive
	m3U CD3	262	130	4.696	2	380	10	5	Positive
	m5C CD3	261	129	2.702	2	380	10	5	Positive
	m2A CD3	285	153	8.08	1	380	10	5	Positive
	Gm CD3	301	152	5.133	1	380	10	5	Positive
	m1G CD3	301	169	5.08	1	380	10	5	Positive
	m5U CD3	262	130	3.915	1	380	10	5	Positive
	Cm CD3	261	112	3.347	3	380	10	5	Positive
	m5s2U CD3	278	146	6.172	2	380	10	5	Positive
	Am CD3	285	136	7.257	1	380	10	5	Positive
	Um CD3	262	113	4.49	1	380	10	5	Positive
	ms2i6A CD3	385	253	10	20	380	10	5	Positive
	mnm5U CD3	291	159	1.428	1	380	10	5	Positive

SILIS	C SILIS	256	119	1.73	1	380	40	5	Positive
	U SILIS	256	119	1.876	1	380	40	5	Positive
	G SILIS	299	162	3.718	3	380	40	5	Positive
	A SILIS	283	146	5.711	1	380	40	5	Positive
	ms2C SILIS	287	150	5.533	3	380	40	5	Positive
	m1A SILIS	298	161	1.745	2	380	10	5	Positive
	m3C SILIS	271	134	1.752	1	380	10	5	Positive
	i6A SILIS	356	219	14.134	1	380	10	5	Positive
	m7G SILIS	314	177	2.021	2	380	10	5	Positive
	m6A SILIS	298	161	8.423	1	380	10	5	Positive
	m2G SILIS	312	175	5.491	2	380	10	5	Positive
	m3U SILIS	271	134	4.696	2	380	10	5	Positive
	m5C SILIS	271	134	2.702	2	380	10	5	Positive
	m2A SILIS	298	161	8.08	1	380	10	5	Positive
	Gm SILIS	314	162	5.133	1	380	10	5	Positive
	m1G SILIS	314	177	5.08	1	380	10	5	Positive
	m5U SILIS	271	134	3.915	1	380	10	5	Positive
	ac4c SILIS	300	163	5.538	2	380	10	5	Positive
	cmnm5U SILI[illegible]	347	210	1.586	1	380	10	5	Positive
	I SILIS	283	146	3.556	1	380	10	5	Positive
	Cm SILIS	271	119	3.347	3	380	10	5	Positive
	m5s2U SILIS	287	150	6.172	2	380	10	5	Positive
	Am SILIS	298	146	7.257	1	380	10	5	Positive
	Psi SILIS	256	220	1.49	1	380	10	5	Positive
	s4U SILIS	272	135	4.753	1	380	10	5	Positive
	Um SILIS	271	119	4.49	1	380	10	5	Positive
	ms2i6A SILIS	403	266	10	20	380	10	5	Positive
	s2C SILIS	272	135	2.288	1	380	10	5	Positive
	t6A SILIS	434	297	7.553	1	380	10	5	Positive
	mnm5U SILIS	302	165	1.491	1	380	10	5	Positive

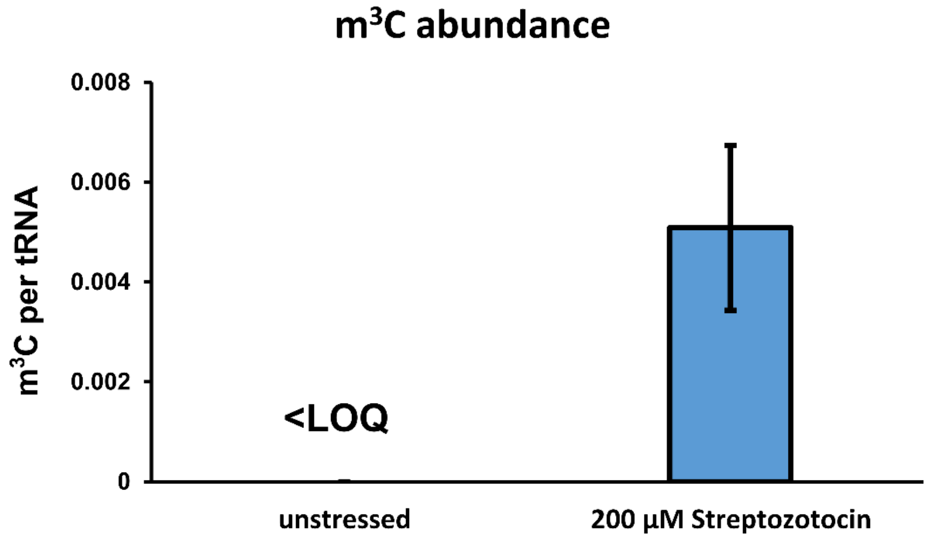

Supplementary Figure 10: m^{3}C abundance in *E. coli* tRNA after incubation with Streptozotocin (200 µM) for 30 min. The signal for m^{3}C in the unstressed samples were below the limit of quantification (LOQ). The experiments were done in biological triplicates and the error bars reflect standard deviation. Source data are provided as a Source Data file.

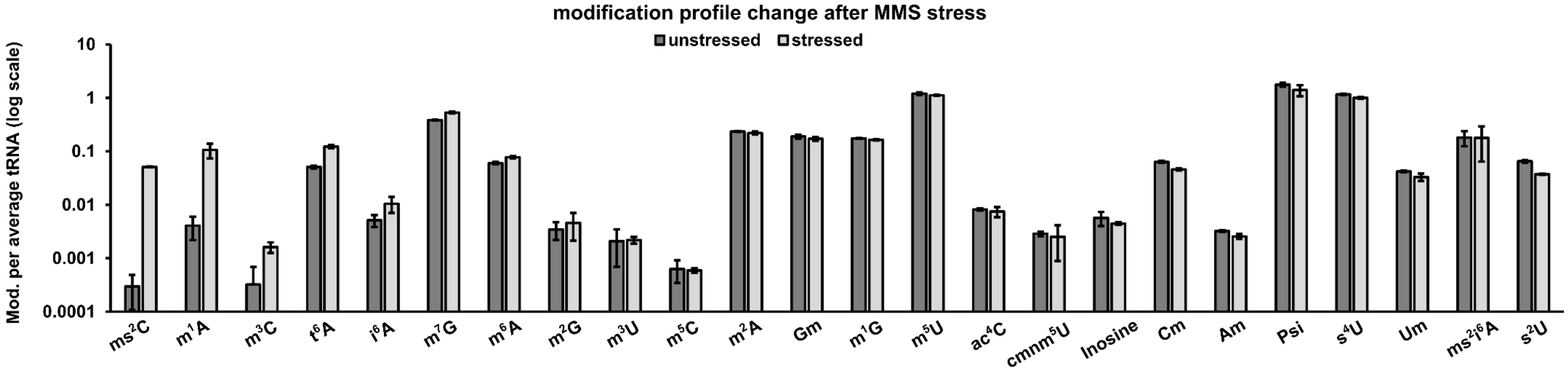

Supplementary Figure 11: Absolute abundance of tRNA modifications in *E. coli* after exposure to 20mM MMS. The dark grey bar shows the modification abundance of an unstressed sample whereas the light grey bar shows the modification abundance after one hour of 20mM MMS stress. From n=3 biol. replicates, error bars reflect standard deviation. Source data are provided as a Source Data file.

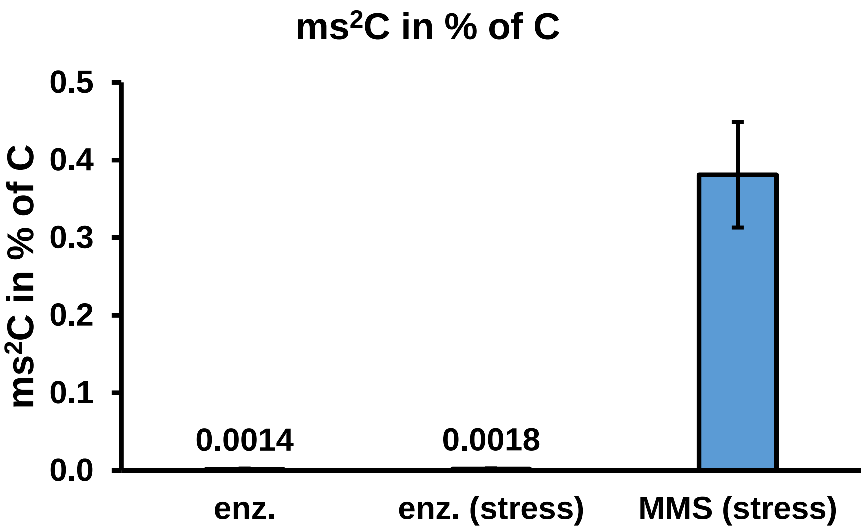

Supplementary Figure 12: NAIL-MS discrimination assay to determine origin of ms^2C *in vivo*. SAM dependent ms^2C per C in % of tRNA from unstressed bacteria (enz.) and 20 mM MMS stressed bacteria (enz. stressed). On the right, the amount of directly methylated ms2C per C in % from 20 mM MMS stressed bacteria is shown. From n=3 biol. replicates, error bars reflect standard deviation. Source data are provided as a Source Data file.

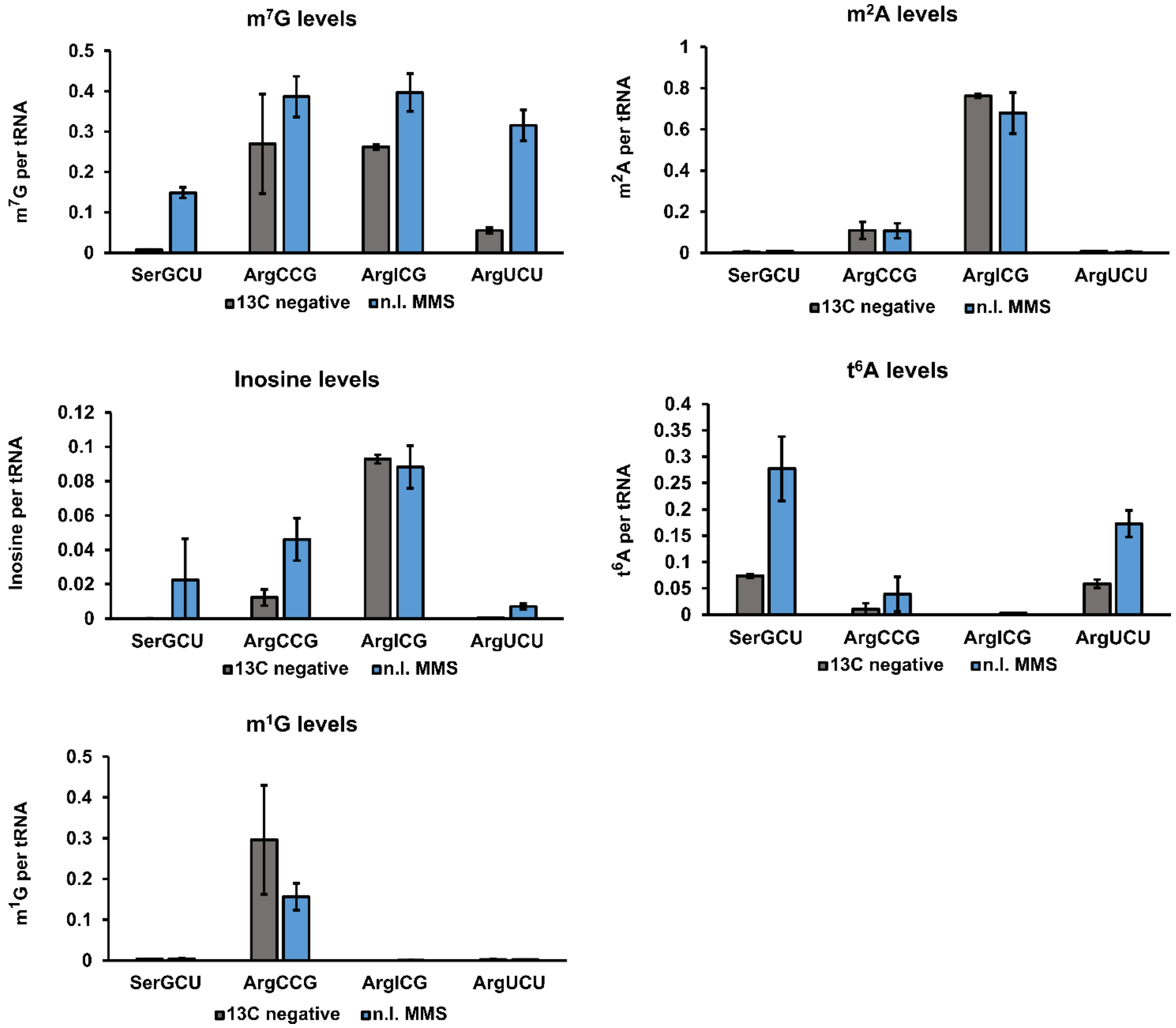

Supplementary Figure 13: Modified nucleoside abundance in comparative NAIL-MS experiment for tRNA SerGCU, ArgCCG, ArgICG and ArgUCU. *E. coli* in the negative control (without MMS) were grown in ^{13}C medium (grey bars, ^{13}C negative) and the 20mM MMS exposed bacteria in non-labeled (blue bars, n.l. MMS) media. The different samples were unified and the tRNA isoacceptors were co-purified in a comparative NAIL-MS experiment as detailed in the text. All experiments are from n=3 biol. replicates and error bars reflect standard deviation. Source data are provided as a Source Data file. The validation of the comparative NAIL-MS experiment is shown in **Supplementary Figure 14**.

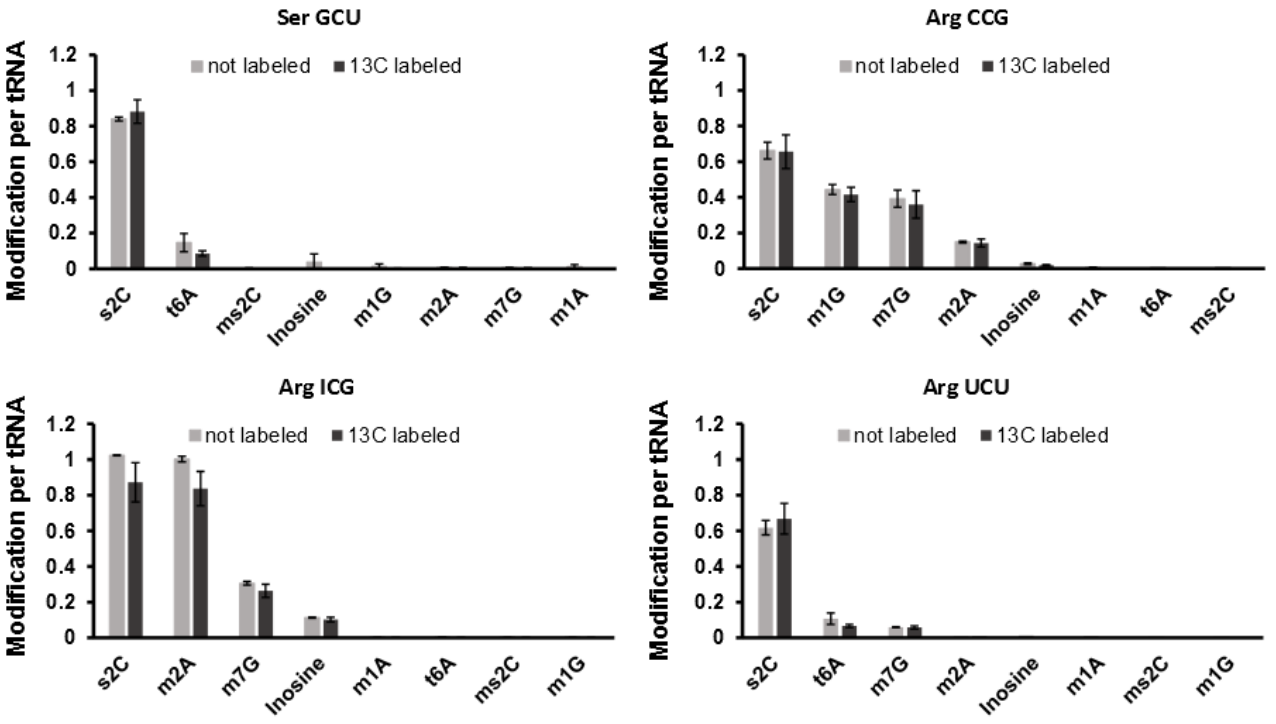

Supplementary Figure 14: Validation experiment of the comparative NAIL-MS experiment The validation is performed by mixing unstressed *E. coli* from unlabeled media and unstressed *E. coli* from carbon-13 labeled media during harvesting. The tRNA and the tRNA isoacceptors from the two *E. coli* cultures are co-purified. The modified nucleosides per tRNA from the unlabeled bacteria (grey bars) and the labeled bacteria (black bars) are of equal abundance. Thus, the validation is considered a success (Reichle *et al.*, Methods, 2018) [4]. From n=3 biol. replicates, error bars reflect standard deviation. Source data are provided as a Source Data file.

Supplementary Table 8: Limit of precision of the comparative NAIL-MS validation shown in **Supplementary Figure 14**. Abbreviation n.d. = not determined All experiments are from n=3 biol. replicates and error bars reflect standard deviation. Source data are provided as a Source Data file.

	upper fold change				lower fold change				standard deviation			
	Ser GCU	Arg CCG	Arg ICG	Arg UCU	Ser GCU	Arg CCG	Arg ICG	Arg UCU	Ser GCU	Arg CCG	Arg ICG	Arg UCU
s2C	1.04162	1.0275	1.1907	1.05574	0.95838	0.97249	0.8093	0.94426	0.08482	0.14137	0.1429	0.16303
ms2C	n.d.	n.d.	n.d.	n.d.	n.d.	n.d.	n.d.	n.d.	n.d.	n.d.	n.d.	n.d.
t6A	1.66802	n.d.	n.d.	1.59955	0.33198	n.d.	n.d.	0.40045	0.31097	n.d.	n.d.	0.41107
Inosine	n.d.	n.d.	1.1135	n.d.	n.d.	n.d.	0.8865	n.d.	n.d.	n.d.	0.1089	n.d.
m1G	n.d.	1.0808	n.d.	n.d.	n.d.	0.91916	n.d.	n.d.	n.d.	0.15722	n.d.	n.d.
m2A	n.d.	n.d.	1.2105	n.d.	n.d.	n.d.	0.7895	n.d.	n.d.	n.d.	0.1114	n.d.
m7G	n.d.	1.1128	1.1776	n.d.	n.d.	0.88715	0.8224	n.d.	n.d.	0.12952	0.1311	n.d.
m1A	n.d.	n.d.	n.d.	n.d.	n.d.	n.d.	n.d.	n.d.	n.d.	n.d.	n.d.	n.d.

Supplementary Table 9: MRM parameters for comparative NAIL-MS analysis.

Compound Group	Compound Name	Precursor Ion (m/z)	Product Ion (m/z)	Ret Time (min)	Delta Ret Time (min)	Fragmentor (V)	Collision Energy (eV)	Cell Accelerator Voltage (V)	Polarity
unlabeled	C	244	112	1.73	1	175	13	5	Positive
	U	245	113	2	2	95	5	5	Positive
	G	284	152	3.718	3	95	17	5	Positive
	A	268	136	5.711	1	110	21	5	Positive
	ms2C	274	142	5.533	3	85	13	5	Positive
	s2C	260	128	2.288	2	85	13	5	Positive
	m1A	282	150	1.745	2	110	21	5	Positive
	m7G	298	166	2.021	2	105	14	5	Positive
	m2A	282	150	8.08	1	125	21	5	Positive
	I	269	137	3.556	1	100	9	5	Positive
	t6A	413	281	7	1	130	9	5	Positive
	m1G	298	166	5.08	1	105	13	5	Positive
13C labeled	C 13C	253	116	1.73	1	175	13	5	Positive
	U 13C	254	117	2	2	95	5	5	Positive
	G 13C	294	157	3.718	3	95	17	5	Positive
	A 13C	278	141	5.711	1	110	21	5	Positive
	ms2C 13C	284	147	5.533	3	85	13	5	Positive
	s2C 13C	269	132	2.288	2	85	13	5	Positive
	m1A 13C	293	156	1.745	2	110	21	5	Positive
	m7G 13C	309	172	2.021	2	105	14	5	Positive
	m2A 13C	293	156	8.08	1	125	21	5	Positive
	I 13C	279	142	3.556	1	100	9	5	Positive
	t6A 13C	428	291	7	1	130	9	5	Positive
	m1G 13C	309	172	5.08	1	105	13	5	Positive
SILIS	C SILIS	256	119	1.73	1	175	13	5	Positive
	U SILIS	256	119	2	2	95	5	5	Positive
	G SILIS	299	162	3.718	3	95	17	5	Positive
	A SILIS	283	146	5.711	1	110	21	5	Positive
	ms2C SILIS	287	150	5.533	3	85	13	5	Positive
	s2C SILIS	272	135	2.288	2	85	13	5	Positive
	m1A SILIS	298	161	1.745	2	110	21	5	Positive
	m7G SILIS	314	177	2.021	2	105	14	5	Positive
	m2A SILIS	298	161	8.08	1	125	21	5	Positive
	I SILIS	283	146	3.556	1	100	9	5	Positive
	t6A SILIS	434	297	7	1	130	9	5	Positive
	m1G SILIS	314	177	5.08	1	105	13	5	Positive

Supplementary Table 10: Primers used for the generation of sfGFP constructs used in this study

gfp_for	CGGAATTCCGCATGAGCAAAGGAGAAGAACTTTTCACTGGAG
gfp_for_AGT	CGGAATTCCGCATGAGTAAAGGAGAAGAACTTTTCACTGGAG
PAS-gfp_for	CGGAATTCCGCATGAGCCCAGCTGCACCTAGCGCTAGCGCAGCAAGCGCACCTAGCGCAGCTAGCAAAGGAGAAGAACTTTTCACTGGAG
PAS-ACT_gfp_for	CGGAATTCCGCATGAGTCCAGCTGCACCTAGTGCTAGTGCAGCAAGTGCACCTAGTGCAGCTAGTAAAGGAGAAGAACTTTTCACTGGAG
gfp_for_TCC	CGGAATTCCGCATGTCCAAAGGAGAAGAACTTTTCACTGGAG
PAS-gfp_for_TCC	CGGAATTCCGCATGTCCCCAGCTGCACCTTCCGCTTCCGCAGCATCCGCACCTTCCGCAGCTTCCAAAGGAGAAGAACTTTTCACTGGAG
gfp_rev	GCTCTAGACTTATTTGTAGAGCTCATCCATGCCATGTG

Full sf*gfp* sequence:

5’AGCAAAGGAGAAGAACTTTTCACTGGAGTTGTCCCAATTCTTGTTGAATTAGATGGTGATGTTAATGGGCACAAATT
TTCTGTCCGTGGAGAGGGTGAAGGTGATGCTACAAACGGAAAACTCACCCTTAAATTTATTTGCACTACTGGAAAACT
ACCTGTTCCGTGGCCAACACTTGTCACTACTCTGACCTATGGTGTTCAATGCTTTTCCCGTTATCCGGATCACATGAAAC
GGCATGACTTTTTCAAGAGTGCCATGCCCGAAGGTTATGTACAGGAACGCACTATATCTTTCAAAGATGACGGGACCT
ACAAGACGCGTGCTGAAGTCAAGTTTGAAGGTGATACCCTTGTTAATCGTATCGAGTTAAAGGGTATTGATTTTAAAG
AAGATGGAAACATTCTTGGACACAAACTCGAGTACAACTTTAACTCACACAATGTATACATCACGGCAGACAAACAAA
AGAATGGAATCAAAGCTAACTTCAAAATTCGCCACAACGTTGAAGATGGTTCCGTTCAACTAGCAGACCATTATCAAC
AAAATACTCCAATTGGCGATGGCCCTGTCCTTTTACCAGACAACCATTACCTGTCGACACAATCTGTCCTTTCGAAAGA
TCCCAACGAAAAGCGTGACCACATGGTCCTTCTTGAGTTTGTAACTGCTGCTGGGATTACACATGGCATGGATGAGCT
CTACAAATAA-3’

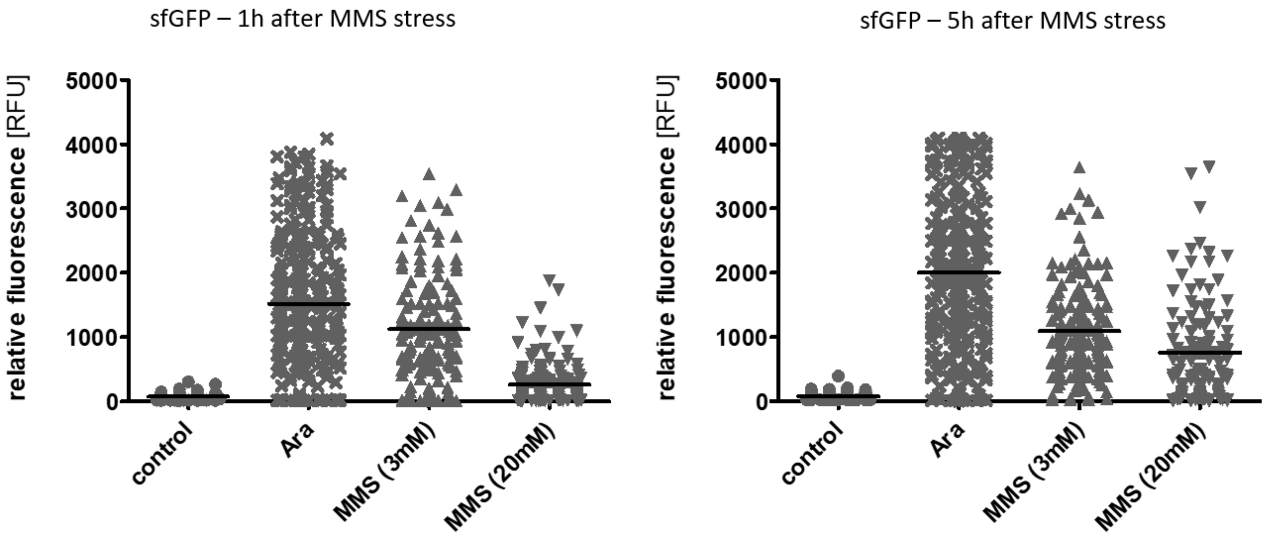

Supplementary Figure 15: Effects of 20 mM or 3 mM MMS stress on sfGFP synthesis in *E. coli* BW25113 cells. Results of single-cell fluorescence microscopy of *E. coli* cells producing sfGFP form the arabinose inducible plasmid pBAD24. Fluorescence was measured 1h or 5h after induction with 0.2% arabinose of non-stressed cells or cells pre-exposed to 20mM or 3 mM MMS stress for 1h. As a control cells were incubated in absence of MMS and arabinose. Black line represents the mean value. Source data are provided as a Source Data file.

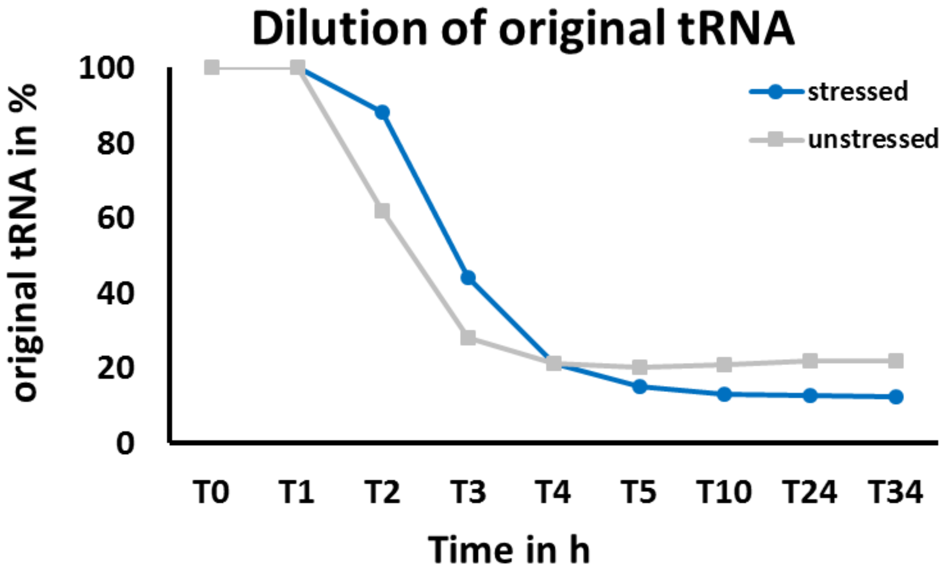

Supplementary Figure 16: RNA dilution curve for total RNA. Comparison of the proportional decrease of original tRNA in comparison to freshly transcribed tRNA during the pulse chase NAIL-MS experiment. The ratio of original (unlabeled canonicals determined by LC-MS) to newly transcribed tRNA (nitrogen-15 labeled canonicals determined by LC-MS) is shown in % original tRNA. blue: from MMS stressed bacteria, grey: from unstressed control bacteria. Mean of n=3 biol. replicates. Source data are provided as a Source Data file.

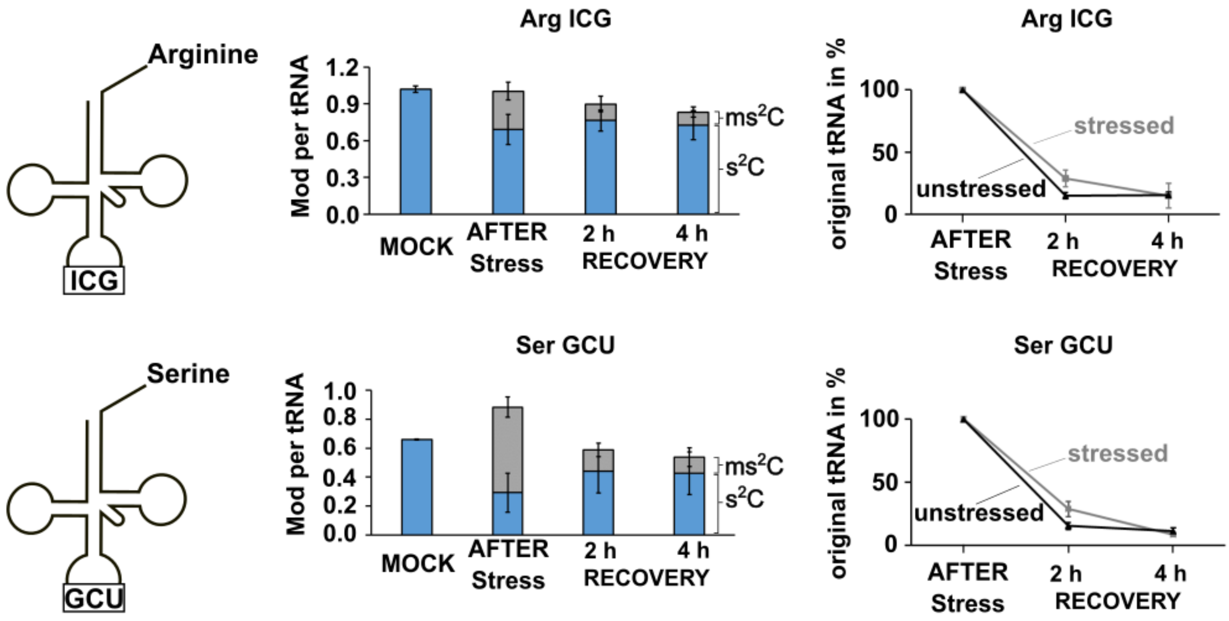

Supplementary Figure 17: Abundance of s^2C (blue) and ms^2C (grey) in $tRNA^{Arg}_{ICG}$ (top) and $tRNA^{Ser}_{GCU}$ (bottom) present during the 20 mM MMS exposure. The bar diagrams show the abundance of the modified nucleosides in original tRNA from a pulse-chase NAIL-MS experiment after 1 h of MMS exposure, and after 2 and 4 hours of recovery. MOCK indicates tRNA from unstressed bacteria. On the right side, the ratio of original to newly transcribed tRNA is shown in % original tRNA. grey: from MMS stressed bacteria, black: from unstressed control bacteria. The isoacceptors were purified from n=3 biol. replicates and error bars reflect standard deviation. Source data are provided as a Source Data file.

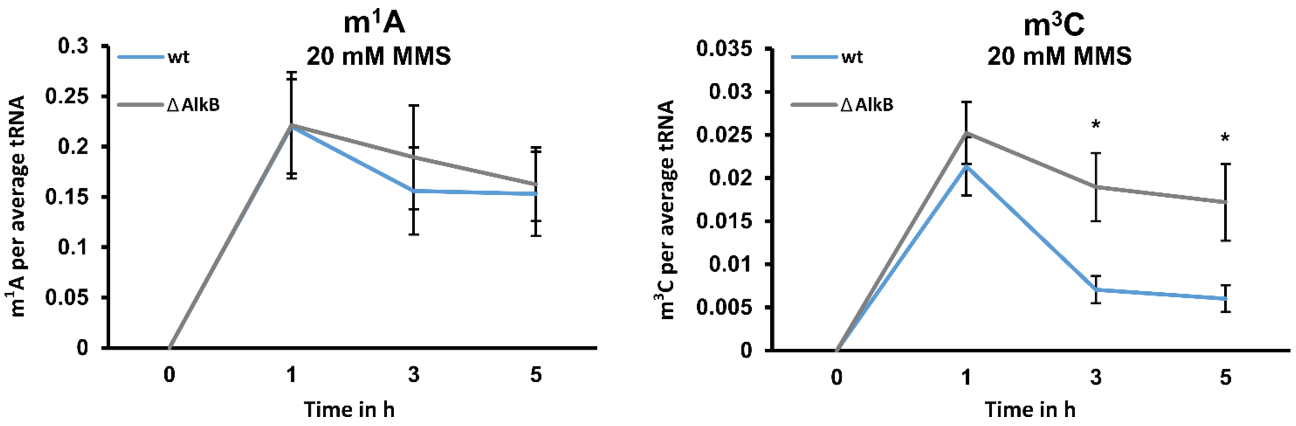

Supplementary Figure 18: Absolute abundance of m^1A and m^3C in wildtype and Δ*alkB E. coli*. Here, the abundance of m^1A and m^3C in original tRNA (distinguished from new transcripts by pulse-chase NAIL-MS as described in **Figure 4a**) in wildtype (wt, light blue) and AlkB deficient *E. coli* (Δ*alkB*, grey) is shown. The bacteria are exposed to 20 mM MMS and harvested directly after the stress (1 h) and after 2 and 4 hours of recovery (3 h, 5 h). All experiments are from n=3 biol. replicates and error bars reflect standard deviation. The statistics were done with student t-test (equal distribution, two-sided): * $p < 0.05$. Source data are provided as a Source Data file.

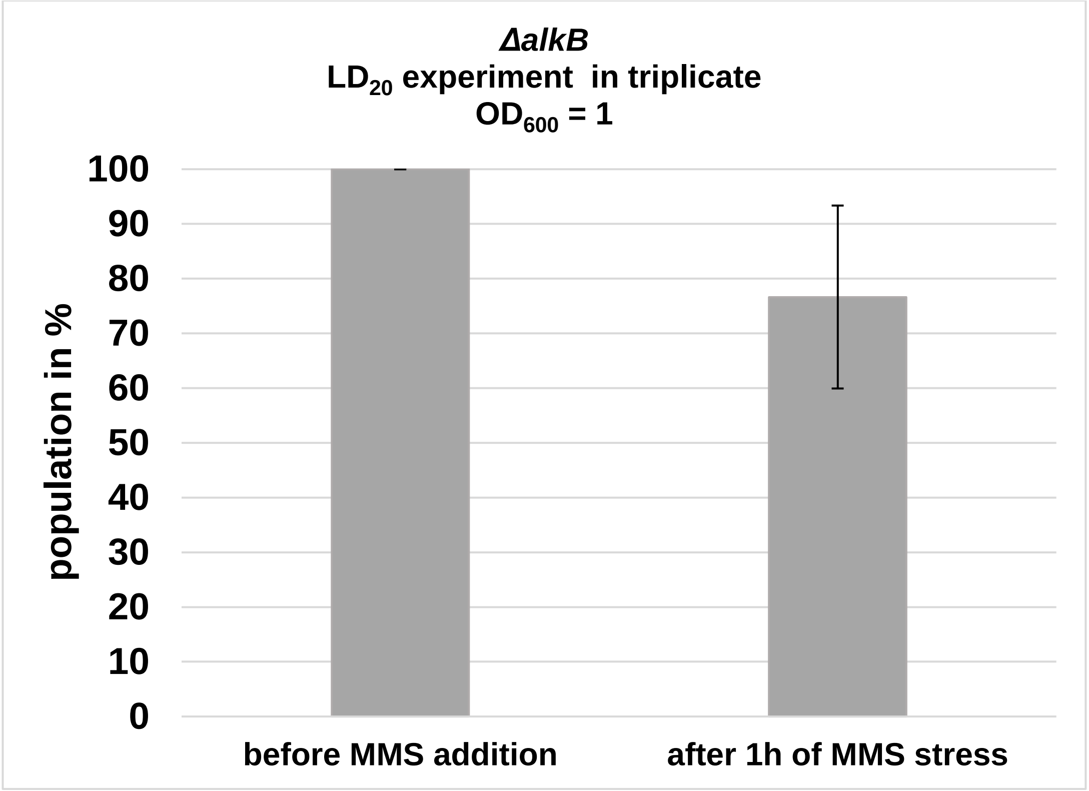

Supplementary Figure 19: Survival assay to determine the lethality of 3mM MMS for the *E.coli ΔalkB* strain Cells were brought to an OD of 1 (error bars reflect the standard deviation of n=3 biol. replicates) and exposed to 3 mM MMS for 60 min.

Supplementary Table 11: MRM parameters for pulse chase NAIL-MS experiments.

Compound Group	Compound Name	Precursor Ion (m/z)	Product Ion (m/z)	Ret Time (min)	Delta Ret Time (min)	Fragmentor (V)	Collision Energy (eV)	Cell Accelerator Voltage (V)	Polarity
unlabeled (original)	C	244	112	1.73	1	175	13	5	Positive
	U	245	113	2	2	95	5	5	Positive
	G	284	152	3.718	3	95	17	5	Positive
	A	268	136	5.711	1	110	21	5	Positive
	ms2C	274	142	5.533	3	85	13	5	Positive
	s2C	260	128	2.288	2	85	13	5	Positive
	m1A	282	150	1.745	2	110	21	5	Positive
	m3C	258	126	1.752	2	88	14	5	Positive
	I	269	137	3.272	2	100	9	5	Positive
	m5U	259	127	3.61	2	145	10	5	Positive
	m2A	282	150	7.85	3	120	20	5	Positive
	m7G	298	166	2.7	2	105	14	5	Positive
	t6A	413	281	6.923	2	130	9	5	Positive
15N/CD3/34S labeled (new)	C 15N	247	115	1.73	1	175	13	5	Positive
	U 15N	247	115	1.876	2	95	5	5	Positive
	G 15N	289	157	3.718	3	95	17	5	Positive
	A 15N	273	141	5.711	1	110	21	5	Positive
	ms2C 34S_CD3_15N	282	150	5.533	3	85	13	5	Positive
	s2C 34S_15N	265	133	2.288	2	85	13	5	Positive
	m1A 15N_CD3	290	158	1.745	2	110	21	5	Positive
	m3C 15N_CD3	264	132	1.752	2	88	14	5	Positive
	I 15N	273	141	3.272	2	100	9	5	Positive
	m5U 15N_CD3	264	132	3.61	2	145	10	5	Positive
	m2A 15N_CD3	290	158	7.85	3	120	20	5	Positive
	m7G 15N_CD3	306	174	2.7	2	105	14	5	Positive
	t6A 15N	419	287	6.923	2	130	9	5	Positive
34S labeled (turnover)	ms2C 34S	276	144	5.533	3	85	13	5	Positive
	s2C 34S	262	130	2.288	2	85	13	5	Positive
SILIS	C SILIS	256	119	1.73	1	175	13	5	Positive
	U SILIS	256	119	1.876	2	95	5	5	Positive
	G SILIS	299	162	3.718	3	95	17	5	Positive
	A SILIS	283	146	5.711	1	110	21	5	Positive
	ms2C SILIS	287	150	5.533	3	85	13	5	Positive
	s2C SILIS	272	135	2.288	2	85	13	5	Positive
	m1A SILIS	298	161	1.745	2	110	21	5	Positive
	m3C SILIS	271	134	1.752	2	88	14	5	Positive
	I SILIS	283	146	3.272	2	100	9	5	Positive
	m5U SILIS	271	134	3.61	2	145	10	5	Positive
	m2A SILIS	298	161	7.85	3	120	20	5	Positive
	m7G SILIS	314	177	2.7	2	105	14	5	Positive
	t6A SILIS	434	297	6.923	2	130	9	5	Positive

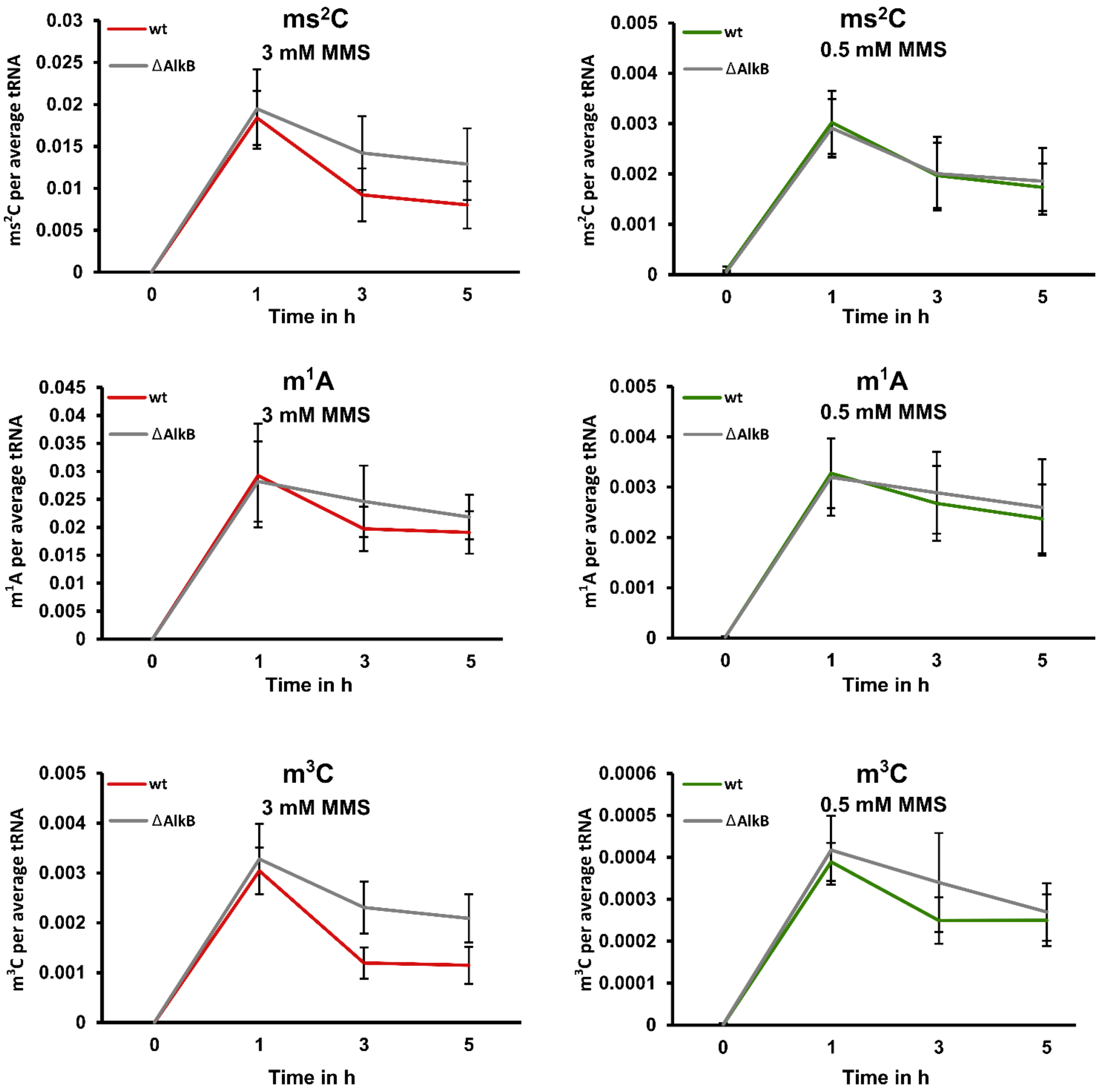

Supplementary Figure 20: Absolute abundance of ms^{2}C, m^{1}A and m^{3}C at different MMS concentrations in WT and Δ*alkB E. coli*. The experimental set up as described in **Figure 4a** was used to investigate the stress behavior at lower MMS concentrations (3mM and 0.5 mM). The bacteria are exposed to MMS and are harvested directly after the stress (1 h) and after 2 and 4 hours of recovery (3 h, 5 h). All experiments are from n=3 biol. replicates and error bars reflect standard deviation. Source data are provided as a Source Data file.

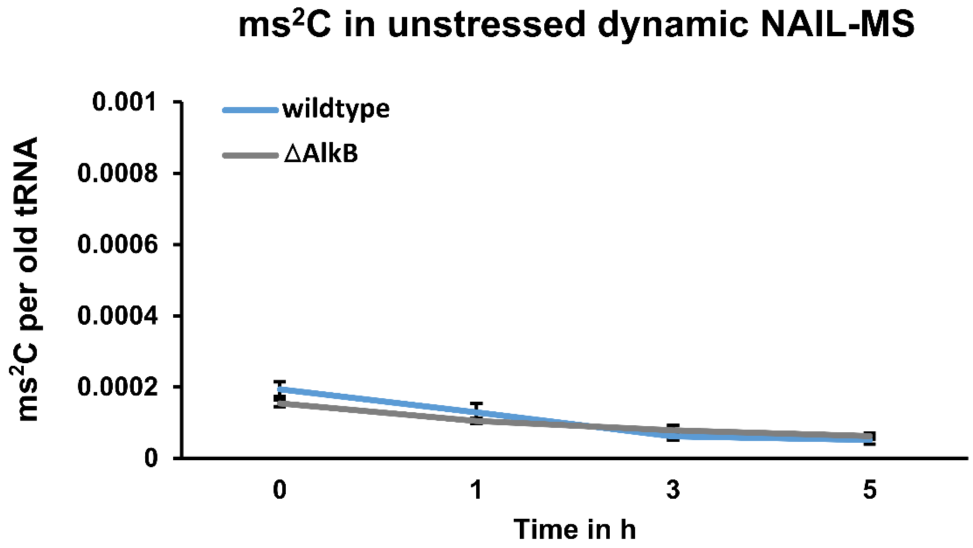

Supplementary Figure 21: Pulse chase control experiment with unstressed *E. coli* WT compared to unstressed Δ*alkB* knockout cells. ms^2C was not observed to accumulate in the Δ*alkB* knockout. The experimental set up is shown in **Figure 4a** in the manuscript. Experiments were done in biological triplicates and error bars reflect standard deviation. Source data are provided as a Source Data file.

Supplementary Table 12: Formula for absolute quantification of unmodified and modified nucleosides from LC-MS/MS signals (area) of a NAIL-MS Pulse Chase experiment and subsequent reference of the modifications to the respective canonicals. As an example, here we chose 2-thiocytidine which was referenced to its canonical nucleoside, cytidine.

	s^2C (fmol)	C (fmol)	Normalization
(1) original	$\frac{\text{area s2C (unlabeled)}}{\text{rRFN s2C} \times \text{area s2C (SILIS)}}$	$\frac{\text{area C (unlabeled)}}{\text{rRFN C} \times \text{area C (SILIS)}}$	$\frac{\text{s2C (original)}}{\text{C (original)}}$
(2) new	$\frac{\text{area s2C (34S, 15N, CD3)}}{\text{rRFN s2C} \times \text{area s2C (SILIS)}}$	$\frac{\text{area C (15N)}}{\text{rRFN C} \times \text{area C (SILIS)}}$	$\frac{\text{s2C (new)}}{\text{C (new)}}$

Supplementary References

1 Baba, T. *et al.* Construction of Escherichia coli K-12 in-frame, single-gene knockout mutants: the Keio collection. *Molecular systems biology* **2**, 2006.0008, doi:10.1038/msb4100050 (2006).

2 Boccaletto, P. *et al.* MODOMICS: a database of RNA modification pathways. 2017 update. *Nucleic acids research* **46**, D303-d307, doi:10.1093/nar/gkx1030 (2018).

3 Kitagawa, M. *et al.* Complete set of ORF clones of Escherichia coli ASKA library (a complete set of E. coli K-12 ORF archive): unique resources for biological research. *DNA research : an international journal for rapid publication of reports on genes and genomes* **12**, 291-299, doi:10.1093/dnares/dsi012 (2005).

4 Reichle, V. F. *et al.* Surpassing limits of static RNA modification analysis with dynamic NAIL-MS. *Methods (San Diego, Calif.)* **156**, 91-101, doi:10.1016/j.ymeth.2018.10.025 (2019).
