# Supplementary material for: NAIL-MS reveals the repair of 2-methylthiocytidine by AlkB in E. coli
Source: Nat Commun. 2019 Dec 6;10:5600. doi: 10.1038/s41467-019-13565-9 (PMC6898146; doi:10.1038/s41467-019-13565-9)
Supplement: Supplementary file 2 — Peer Review [file 41467_2019_13565_MOESM2_ESM.pdf]

Editorial Note: This manuscript has been previously reviewed at another journal that is not operating a transparent peer review scheme. This document only contains reviewer comments and rebuttal letters for versions considered at *Nature Communications.*

REVIEWERS' COMMENTS:

Reviewer #1 (Remarks to the Author):

My comments at this stage relate only to the translation assays presented in the revised manuscript, that are important to establish that ms2C formation negatively affects translation.

The authors do not show the ttC KO data, so it is difficult to evaluate properly. Nevertheless, if the authors' model holds (namely that s2C is a major target of MMS alkylation and negatively affects translation), one prediction would be that the detrimental effect of MMS treatment would be mitigated in the KO -- that is, the relative impairment in translation compared to unstressed KO cells would be smaller than what is observed in the WT background. Again, it is unclear if this is the case since the data is not shown.

As to the codon replacement assay, I am somewhat surprised that the authors claim that ms2C formation is the "only explanation" for the translational impairment observed. It is uncommon to see that type of statement in the the evaluation of complex biological phenomena. Particularly, in this case, when the experiments are not being conducted with a precise tool for modulating the levels of the modification, and only one codon replacement has been performed and evaluated.

The data would be much more compelling if it was supplemented with in vitro data showing that a synthetic ms2C-containing tRNA functions less efficiently in a translational assay, as well as the inclusion of additional examples of codon replacement in vivo that produce the same effect.

In the absence of this data, I would recommend that the conclusions relating to the specific effect of ms2C (rather than simply MMS) on translation be significantly softened.

REVIEWERS' COMMENTS:

Reviewer #1 (Remarks to the Author):

My comments at this stage relate only to the translation assays presented in the revised manuscript, that are important to establish that ms2C formation negatively affects translation.

The authors do not show the ttC KO data, so it is difficult to evaluate properly. Nevertheless, if the authors' model holds (namely that s2C is a major target of MMS alkylation and negatively affects translation), one prediction would be that the detrimental effect of MMS treatment would be mitigated in the KO -- that is, the relative impairment in translation compared to unstressed KO cells would be smaller than what is observed in the WT background. Again, it is unclear if this is the case since the data is not shown.

As to the codon replacement assay, I am somewhat surprised that the authors claim that ms2C formation is the "only explanation" for the translational impairment observed. It is uncommon to see that type of statement in the the evaluation of complex biological phenomena. Particularly, in this case, when the experiments are not being conducted with a precise tool for modulating the levels of the modification, and only one codon replacement has been performed and evaluated.

The data would be much more compelling if it was supplemented with in vitro data showing that a synthetic ms2C-containing tRNA functions less efficiently in a translational assay, as well as the inclusion of additional examples of codon replacement in vivo that produce the same effect.

In the absence of this data, I would recommend that the conclusions relating to the specific effect of ms2C (rather than simply MMS) on translation be significantly softened.

We thank the reviewer for reviewing our manuscript and his/her expert remarks. In light of the raised comments, we have revised the in vivo translation section of the manuscript. We agree with the reviewer that all data should be shown. While waiting for the review, we have done additional replicates of the WT and ttcA KO experiment but the results remain inconclusive. We have removed all statements made on these experiments. Thus we agree with the reviewer and we have significantly softened the statements about the impact of ms2C on translation. As suggested by the reviewer, future in vitro translation assays alongside structural analysis of the damaged tRNA will elucidate the impact of ms2C on translation in a less complex environment.
